# A mechanism for pathological oscillations in mouse retinal ganglion cells in a model of night blindness

**DOI:** 10.1085/jgp.202413749

**Published:** 2025-10-16

**Authors:** Sho Horie, Konan Sakuta, Keigo Tada, Haruki Tokumoto, Taketo Nishimoto, Katsunori Kitano, Masao Tachibana, Chieko Koike

**Affiliations:** 1 Graduate School of Pharmacy, Ritsumeikan University, Kusatsu, Japan; 2 Graduate School of Information Science and Engineering, Ritsumeikan University, Ibaraki, Japan; 3 College of Information Science and Engineering, Ritsumeikan University, Ibaraki, Japan; 4 Center for Systems Vision Science, Research Organization of Science and Technology, Ritsumeikan University, Kusatsu, Japan; 5 Ritsumeikan Global Innovation Research Organization (R-GIRO), Ritsumeikan University, Kusatsu, Japan; 6 College of Pharmaceutical Sciences, Ritsumeikan University, Kusatsu, Japan

## Abstract

TRPM1 channels, regulated by mGluR6 at the dendrites of retinal ON bipolar cells (BCs), play a crucial role in visual signal transduction. Both *Trpm1* knockout (KO) and *mGluR6* KO mice are models of congenital stationary night blindness with grossly normal morphology. However, robust pathological spontaneous oscillations in retinal ganglion cells (RGCs) have been observed in *Trpm1* KO retinas but not in *mGluR6* KO retinas. We investigated the mechanism underlying these oscillations in the *Trpm1* KO retina using whole-cell clamp techniques. We found that inhibitory and excitatory synaptic inputs produced anti-phase oscillations in OFF and ON RGCs, respectively, and that oscillations could be suppressed by blockers targeting the AII amacrine cell (AC) pathway. The *rd1* retina, a model for retinitis pigmentosa with severe photoreceptor degeneration, displays similar oscillations to the *Trpm1* KO retina. Morphological and immunohistochemical analyses revealed similar alterations in the *Trpm1* KO and *rd1* retinas when compared to the *mGluR6* KO and wild-type retinas: namely, rod BCs (RBCs) in both *Trpm1* KO and *rd1* retinas showed reduced dendritic TRPM1 labeling and smaller axon terminals. Furthermore, RBCs in the *Trpm1* KO retina were significantly hyperpolarized. *In silico* simulation of the BC-AII AC-RGC network suggests that the reduction of RBC and ON cone BC outputs to AII ACs contributes to RGC oscillations. Our findings suggest that TRPM1 deficiency in ON BCs produces RGC oscillations in association with RBC axon remodeling and reduced ON BC outputs, and may represent a shared circuit mechanism underlying pathological oscillations across different causes of outer retinal diseases.

## Teaser

Horie et al. show that TRPM1 deficiency in ON bipolar cells produces retinal ganglion cell oscillations in association with rod bipolar cell axon remodeling and reduced ON bipolar cell outputs, and may represent a shared circuit mechanism underlying pathological oscillations across different causes of outer retinal diseases.

## Introduction

Retinitis pigmentosa (RP), an inherited disorder characterized by progressive degeneration of photoreceptors, has been studied using model mice with retinal degeneration (*rd*) (Grover et al., 1999; Hartong et al., 2006; Margolis et al., 2008; Goo et al., 2011). In the case of *rd1* mouse, rods are almost completely lost by P30–P40 (Carter-Dawson et al., 1978; Jiménez et al., 1996). Retinal ganglion cells (RGCs) of *rd* mice show pathological oscillatory spontaneous firing (Margolis et al., 2008, 2014; Stasheff, 2008; Borowska et al., 2011; Menzler and Zeck, 2011; Yee et al., 2012; Choi et al., 2014; Poria and Dhingra, 2015). The oscillations in postsynaptic RGCs are driven by oscillations in presynaptic AII amacrine cells (ACs) (Borowska et al., 2011; Trenholm et al., 2012; Choi et al., 2014; Margolis et al., 2014). RGC oscillations, often referred to as “noise,” may disrupt visual information processing. Thus, understanding the mechanisms underlying RGC oscillations is crucial for finding effective therapeutic strategies for RP.

It is widely assumed that RGC oscillations in the *rd1* mouse retina may be caused primarily by photoreceptor degeneration, and that the following AII AC oscillations may be induced by intrinsic membrane properties of AII ACs (Choi et al., 2014) or by network properties of AII ACs and ON cone bipolar cells (ON CBCs) (Trenholm et al., 2012). In addition to RGC oscillations, the *rd1* mouse retina undergoes significant anatomical remodeling. The sprouting neurites from cones develop ectopic synapses with rod bipolar cell (RBC) soma (Haq et al., 2014). Horizontal cells also extend abnormal processes toward the inner plexiform layer (IPL), whereas the dendrites of ON BCs retract (Strettoi and Pignatelli, 2000; Strettoi et al., 2002, 2003; Chen et al., 2012). Various forms of anatomical remodeling hinder our understanding of how photoreceptor degeneration leads to oscillations in the inner retina of the *rd* mice.

In the present study, we focus on the *Trpm1* knockout (KO) mouse, a model for congenital stationary night blindness (CSNB). TRPM1, a member of the transient receptor potential cation channel subfamily M, is a critical visual transduction channel regulated by mGluR6 on the dendrites of ON BCs. Both *Trpm1* KO and *mGluR6* KO mice serve as models of CSNB and exhibit a loss of ON responses despite grossly normal retinal structures (Masu et al., 1995; Koike et al., 2009; Morgans et al., 2009; Koike et al., 2010; Nakamura et al., 2010; Xu et al., 2012). However, a notable difference is that pathological spontaneous RGC oscillations are prominently observed in the *Trpm1* KO mouse, whereas oscillations are infrequent or absent in the *mGluR6* KO mouse (Takeuchi et al., 2018; Hasan et al., 2020). Oscillatory activity occurs in other CSNB models, such as *Nyx*^*nob*^, *Lrit3*^*emrgg1*^, and *Cav1.4* KO mice (Hasan et al., 2020; Hölzel et al., 2023). Notably, disruption of these genes leads to impaired membrane localization of TRPM1 (Pearring et al., 2011; Neuillé et al., 2015; Maddox et al., 2020). In particular, *Nyx* and *Lrit3* play crucial roles in ensuring proper membrane targeting of TRPM1 (Pearring et al., 2011; Neuillé et al., 2015). Taken together, these consistent associations raise the possibility that TRPM1 loss is a key contributing factor in generating oscillatory activity in CSNB—a hypothesis we investigate using *Trpm1* KO mouse retinas.

In this study, we used whole-cell recordings, morphological and immunohistochemical analyses, and computational modeling to investigate the mechanisms underlying RGC oscillations in the *Trpm1* KO mouse retina. We compared *Trpm1* KO and *rd1* mouse retinas and observed shared morphological alterations in RBCs. Using *in silico* models, we examined how TRPM1 loss and these morphological alterations might together contribute to the emergence of oscillatory activity.

## Materials and methods

### Animals

All animal experimental protocols were approved by the Animal Research Committee of Ritsumeikan University and conducted in accordance with local guidelines and the ARVO Statement for the Use of Animals in Ophthalmic and Vision Research. For electrophysiological experiments, 1- to 2-mo-old wild-type (WT) and *Trpm1* KO mice (129 Sv/Ev background) were used. For immunohistochemical experiments, 1-mo-old WT, *Trpm1* KO, and *mGluR6* KO mice (129 Sv/Ev background), as well as 1-mo-old WT, *mGluR6* KO, and *Pde6b*^*rd1-2J*^/J (stock #004766; JAX, referred to as *rd1*) mice (C57BL/6J background), were used. Mice of either sex were used. The *Trpm1* KO mouse line, generated by homologous recombination–mediated disruption of the *Trpm1* gene, was provided by Dr. Takahisa Furukawa, Osaka University, Suita, Japan. The *mGluR6* KO mouse line, generated by homologous recombination–mediated disruption of the *mGluR6* gene, was provided by Dr. Shigetada Nakanishi, Kyoto University, Kyoto, Japan. The *rd1* mouse retinas were provided by Dr. Michiko Mandai, Research Center, Kobe City Eye Hospital, Kobe, Japan. Mice were housed in a temperature-controlled room under a 12-h light/12-h dark cycle. Fresh water and diet were always available.

### Flat-mount preparation

Mice were dark-adapted overnight prior to electrophysiological experiments. Each mouse was sacrificed by cervical dislocation under a dim red light, and eyes were enucleated. Under a stereomicroscope equipped with an infrared (IR) image converter (V6833P; Hamamatsu Photonics) and IR illumination (HVL-IRM; Sony), retinas were dissected in a dish filled with Ames’ medium (A1372; US Biological or A1420; Sigma-Aldrich) bubbled with 95% O_2_/5% CO_2_ at room temperature. The retina isolated from the sclera and pigment epithelium was transferred to a recording chamber, and flat-mounted with the ganglion cell layer (GCL) facing upward. The retina was anchored by a platinum horseshoe with fine nylon threads.

### Slice preparation

Mice were dark-adapted overnight prior to the procedure. Using an IR image converter and IR illumination, we performed retinal dissection in ice-cold HEPES-buffered solution (130 mM NaCl, 10 mM HEPES, 2.5 mM KCl, 2.5 mM CaCl_2_, 1 mM MgCl_2_, 28 mM glucose, pH 7.4, with NaOH) continuously bubbled with 100% O_2_. Following enucleation, the cornea and lens were carefully removed to create an eyecup. The retina, with the attached retinal pigment epithelium (RPE) and sclera, was cut into quarters and placed RGC side down onto a membrane filter (GSWP04700; Millipore). The RPE and sclera were then gently peeled away from the retina. The retina was subsequently sliced into 200-µm-thick sections using a tissue slicer (EDMS14-234; NARISHIGE). Individual sections were secured in the recording chamber using a platinum horseshoe with fine nylon threads.

### Whole-cell recordings

Whole-cell clamp recordings were performed from αRGCs (soma diameter >15 µm) in the flat-mount preparation and from RBCs in the retinal slice preparation. Patch pipettes were filled with either K^+^-based internal solution (125 mM K gluconate, 10 mM KCl, 10 mM HEPES, 10 mM phosphocreatine, 0.5 mM EGTA, 0.05 mM CaCl_2_, 2 mM MgCl_2_, 5 mM ATP-Na_2_, 0.5 mM GTP-Na_3_, and 0.2% neurobiotin [NB], pH 7.4, with KOH) for current-clamp recordings in RGCs, or Cs^+^-based internal solution (102 mM CsMeSO_3_, 20 mM HEPES, 10 mM phosphocreatine, 5 mM EGTA, 0.5 mM CaCl_2_, 2.5 mM MgCl_2_, 5 mM ATP-Na_2_, 0.5 mM GTP-Na_3_, 5 mM QX-314, and 0.2% NB, pH 7.4, with CsOH [E_Cl_ ∼ −60 mV]) for voltage-clamp recordings in RGCs. For RBC recordings, only the K^+^-based internal solution was used, in which 50 µM sulforhodamine was included instead of NB.

Recordings were obtained using an EPC 10 amplifier (HEKA; Multi Channel Systems MCS GmbH) controlled by PATCHMASTER software (version 2.73.5; HEKA). Current and voltage records were sampled at 16 kHz and low-pass–filtered at 2.9 kHz. Patch pipettes were pulled from borosilicate glass capillaries (CNC 1.5; Ken Enterprise, or B150-86-10; Sutter Instrument), using a puller (P97; Sutter Instrument). The resistance of pipettes in Ames’ medium was 4–9 MΩ for αRGCs and 9–15 MΩ for RBCs. A correction was made for liquid junction potential (∼10 mV). The retina was continuously superfused with Ames’ medium bubbled with 95% O_2_/5% CO_2_ at the rate of 2.5 ml/min at 32°C.

### Light stimulation

In recordings from flat-mount preparation, a light stimulus was projected from a DLP projector (L51W, refresh rate 60 Hz, 1,280 × 1,024 pixels; NEC), in which the projection lens was replaced with an achromatic lens (f = 120 mm). The image was focused onto the photoreceptor layer through an objective lens (4× Plan Achromat; Nikon), after removing the condenser lens of an upright microscope (Axio Examiner D1; Zeiss). The retina was stimulated with a white spot (diameter: 300 µm) for 2 s (0.26 cd/m^2^) on a dark background (0.0012 cd/m^2^) every 10 s. Luminance was measured by a luminance meter (CS-150; KONICA MINOLTA).

In recordings from slice preparation, retinal images were observed using the upright microscope with IR differential interference contrast (DIC) optics.

### Cell identification

Following recordings, retinas were fixed in 4% paraformaldehyde in 0.1 M phosphate buffer for 1 h at room temperature. They were then rinsed several times in phosphate-buffered saline (PBS) and incubated overnight at 4°C in a blocking solution (4% normal donkey serum, 0.5% Triton X-100, and 0.1% sodium azide in PBS). Retinas were subsequently incubated with primary antibodies in the blocking solution for 7 days at 4°C. After washing with PBS, retinas were incubated with secondary antibodies for 2 days at 4°C. After washing with PBS, retinas were mounted onto microscope slides. Primary antibodies were goat anti-choline acetyltransferase (ChAT, 1:100, AB144P; Sigma-Aldrich) and mouse anti-SMI-32 (1:1,000, 801701; BioLegend). Secondary antibodies were streptavidin-Alexa 488 (1:500, S32354; Invitrogen), donkey anti-goat Alexa 568 (1:500, A-11057; Invitrogen), and donkey anti-mouse Alexa 647 (1:500, A-31571; Invitrogen).

Z-stack images (1,024 × 1,024 pixels, 0.3 µm/slice) were acquired by a confocal microscope (LSM900; Zeiss) equipped with 40× objective lens (Plan-Apochromat 40×/0.95 Corr M27; Zeiss). Images were analyzed using ImageJ (version 1.53t; National Instutes of Health) and custom codes written in Python. Among recorded RGCs, SMI-32–positive cells with a large soma size (>15 μm) were identified as αRGCs (Krieger et al., 2017). These αRGCs were further subclassified into OFF or ON types based on their dendritic stratification relative to ChAT bands in the IPL.

The cell intracellularly filled with sulforhodamine was identified as RBCs based on the soma location within the inner nuclear layer (INL) and on the position and morphology of axon terminals in the IPL.

### Pharmacology

Pharmacological agents dissolved in Ames’ medium were bath-applied: DNQX (100 µM; Sigma-Aldrich) for blocking AMPA/KA glutamate receptors; d-AP5 (50 µM; Tocris Bio-Techne) for blocking NMDA glutamate receptors; MFA (100 µM; Sigma-Aldrich) for blocking gap junctions; strychnine (10 µM; Sigma-Aldrich) for blocking glycine receptors.

### Data analysis

All data analyses were performed using Python. The power spectral density (PSD) was calculated from the autocorrelogram of spike events recorded during a 145-s membrane potential trace under the current-clamp mode. For the PSD and the autocorrelogram, 0.5-Hz bin width and 1-ms bin width were used, respectively. Oscillation frequency was defined as the frequency corresponding to the peak power between 1.5 and 30 Hz in the PSD. The cross-correlogram (CCR) was calculated based on the spike timing between a pair of RGCs. The time lag was converted to phase by referencing the fundamental frequency of oscillations in each RGC pair.

The amplitude of spontaneous synaptic currents under the voltage-clamp mode was analyzed as follows. The current trace was band-pass–filtered (Bessel filter; 1–100 Hz), and the trace during the last 2.5 s of a 3-s voltage step was used to generate a current histogram with 5-pA bin width. A cumulative distribution function (CDF) was then calculated from the histogram. The current amplitude was defined as the distance between 0.05 and 0.95 in the CDF. The polarity of synaptic current was determined by the value of skewness of the current histogram (i.e., the inward and outward currents were less than −0.15 and >0.15, respectively, whereas the values in between were not used for analysis). The reversal potential of the synaptic current was estimated from the current amplitude–voltage relationship. The PSD of spontaneous synaptic current was calculated using a bin width of 0.5 Hz from the band-pass–filtered trace.

The membrane potential of RBCs was recorded for 40 s under the current-clamp mode, and the voltage histogram was constructed using a bin width of 1 mV. The resting membrane potential was defined as the membrane potential corresponding to the peak value of the histogram. For voltage-clamp recordings, cells were held at −60 mV and stepped from −80 to 0 mV in +10 mV increments to construct current–voltage (I–V) curves. The slope conductance was subsequently calculated from the linear portion of the I–V curve, specifically near the resting membrane potential.

### Immunohistochemistry

Mice were euthanized by cervical dislocation, and the eyes were enucleated. In a dish filled with PBS, the cornea and lens were removed, and the retinas were isolated from the eyecups. Retinas were fixed in 4% paraformaldehyde in 0.1 M phosphate buffer for 20 min at room temperature. After washing with PBS, the retinas were embedded in 4% low-melting agarose (NIPPON GENE) in PBS. Retinas were sectioned at 50-µm thickness using a vibratome (PRO7; Dosaka EM) and incubated in PBS containing either 5% normal goat serum or 5% normal donkey serum, both with 0.1% Triton X-100, for 1 h at room temperature. After blocking, sections were incubated with primary antibodies overnight at 4°C. After washing with PBS, the sections were then incubated with secondary antibodies. Sections were rinsed several times with PBS and mounted on microscope slides. The primary and secondary antibodies used are listed in Table 1.

**Table 1.** Antibodies

Antibodies	Source	Cat	Dilution
Guinea pig anti-mGluR6	Lab-made (Ueno et al., 2025, *Preprint*)	N/A	1:5,000
Mouse anti-CtBP2	BD Biosciences	612044	1:1,000
Mouse anti-PKCα	Sigma-Aldrich	P5704	1:3,000
Mouse anti-Syt2	Developmental Studies Hybridoma Bank	Znp-1	1:1,000
Sheep anti-TRPM1	Courtesy of Dr. Kirill Martemyanov	N/A	1:500
Rabbit anti-cone arrestin	Sigma-Aldrich	AB15282	1:1,000
Goat anti-GluK1	Courtesy of Dr. Steven H. DeVries, Northwestern University, Evanston, IL, USA	N/A	1:1,000
Goat anti-ChAT	Sigma-Aldrich	AB144P	1:50
Guinea pig anti-VGluT1	Sigma-Aldrich	AB5905	1:6,000
Goat anti-guinea pig IgG-Alexa 488	Invitrogen	A-11073	1:500
Goat anti-mouse IgG-Alexa 555	Invitrogen	A-21425	1:500
Donkey anti-mouse IgG-Alexa 488	Jackson ImmunoResearch	715-545-151	1:500
Donkey anti-sheep IgG-Alexa 555	Invitrogen	A-21436	1:250
Donkey anti-rabbit IgG-Alexa 488	Jackson ImmunoResearch	711-545-152	1:500
Donkey anti-guinea pig IgG-Alexa Cy3	Jackson ImmunoResearch	706-165-148	1:500
Donkey anti-goat IgG-Alexa 647	Abcam	ab150139	1:500
Donkey anti-mouse IgG-Alexa 488	Abcam	ab181289	1:500
Donkey anti-mouse IgG-Alexa 647	Molecular Probes	A31571	1:500
Donkey anti-goat IgG-Alexa 568	Invitrogen	A-11057	1:500
DAPI	Sigma-Aldrich	D8417	1:10,000

Z-stack images (1,024 × 1,024 pixels, 0.2 µm/slice) were acquired by the confocal microscope equipped with a 63× oil immersion lens (Plan-Apochromat 63×/1.4 Oil DIC M27, Zeiss). Voxel size was 0.1 × 0.1 × 0.2 µm per pixel. Image analyses were performed using ImageJ, Imaris (ver. 9.9.1; Oxford Instruments), and Python. The volume of RBC axon terminals was measured from the merged volume of PKCα and VGLUT1 signals. The distributions of RBC axon terminals and type 6 BC axon terminals in the IPL were defined as the starting point (INL side) and ending point (GCL side) of PKCα signal distribution and Syt2 signal distribution by eye, and it was evaluated relative to the ChAT bands (OFF band: 0%; ON band: 100%).

### Computer simulations

Retinal neural circuit models were constructed to reproduce not only the normal firing of RGCs in WT retinas but also the spontaneous oscillatory firing of RGCs observed in pathological retinas. The following three steps were involved in the simulation. (1) Construction of a normal retinal neural circuit model (the WT circuit model): based on the experimental data obtained from WT retinas, we constructed a model that responded to both the offset and onset of light stimuli (Dunn et al., 2006; Margolis and Detwiler, 2007; Arman and Sampath, 2012). (2) Application of known pathological conditions: pathological conditions identified in previous studies (Chua et al., 2009; Koike et al., 2009; Križaj et al., 2010; Takeuchi et al., 2018) were applied to the WT mouse model constructed in step 1). Additionally, certain pathological conditions observed in the present study were also incorporated. (3) Exploration of conditions leading to RGC oscillations: based on the pathological model constructed in step 2, we explored the conditions under which RGCs exhibited pathological spontaneous oscillatory firing.

**Figure S1.**
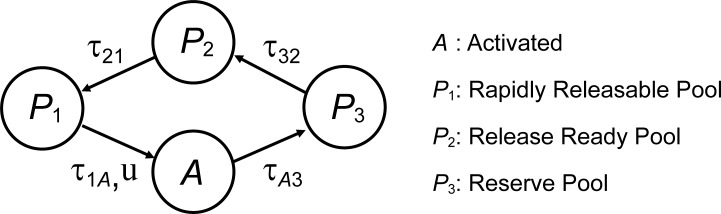
**4-state ribbon synapse model**.

The retinal neural circuit model was implemented using the following neuron and synapse models. *Neuron models*: BCs (RBC, OFF CBC, and ON CBC) (Usui et al., 1996), AII AC (Choi et al., 2014), and RGCs (OFF and ON RGCs) (Fohlmeister et al., 2010). The BC model was simplified from a multicompartment model to a single-compartment model, whereas the other neuron models were used as originally described. *Synapse models*: electrical synapses, conventional synapses, and ribbon synapses. In the ribbon synapse model, synaptic vesicles belonged to one of four states (Fig. S1) and underwent state transitions according to Eqs. 1, 2, 3, 4, and 5.

$$\frac{dP_{3}}{dt}=\frac{A}{\tau_{A3}}-\frac{P_{3}\left(P_{2,\max}-P_{2}\right)}{\tau_{32}}$$ (1)

$$\frac{dP_{2}}{dt}=\frac{P_{3}\left(P_{2,\max}-P_{2}\right)}{\tau_{32}}-\frac{P_{2}\left(P_{1,\max}-P_{1}\right)}{\tau_{21}}$$ (2)

$$\frac{dP_{1}}{dt}=\frac{P_{2}\left(P_{1,\max}-P_{1}\right)}{\tau_{21}}-\frac{uP_{1}}{\tau_{1A}}$$ (3)

$$\frac{dA}{dt}=\frac{uP_{1}}{\tau_{1A}}-\frac{A}{\tau_{A3}}$$ (4)

$$u=\frac{1}{2}\left[1+\tanh\left(\frac{V_{pre}-V_{th}}{V_{slp}}\right)\right]$$ (5)

**Figure S2.**
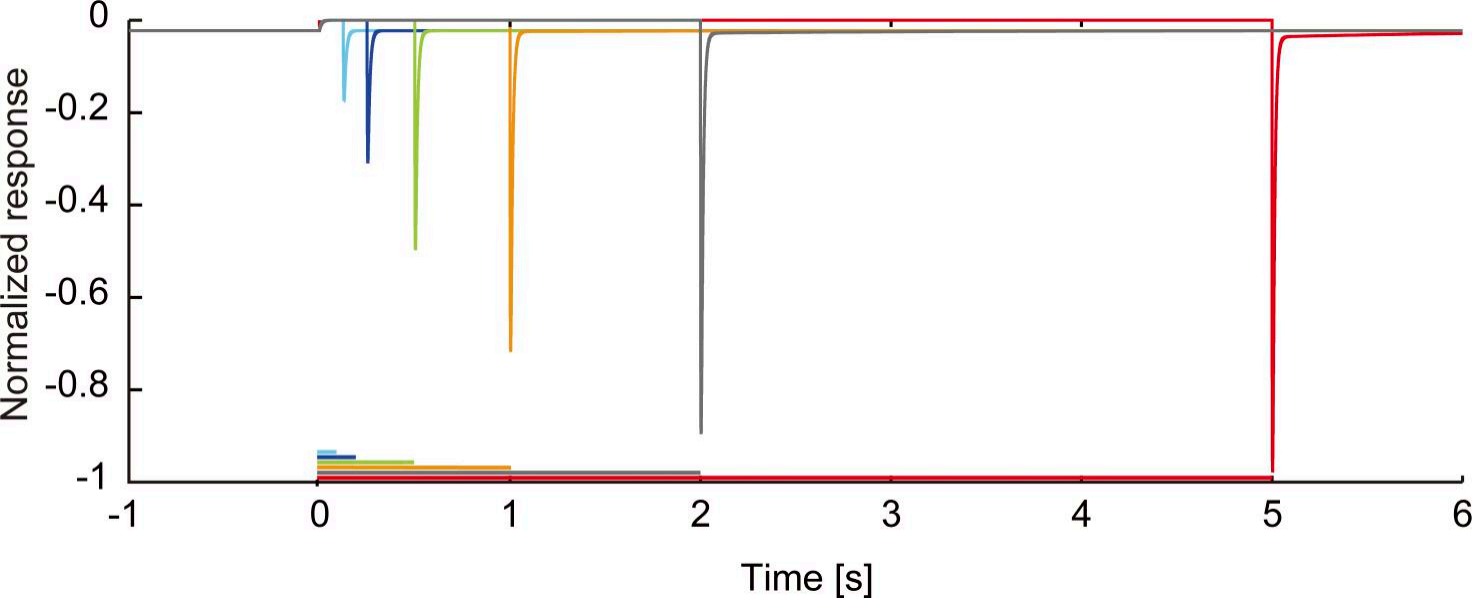
**Response recovery of the ribbon synapse model.** The magnitudes of the responses following the inactivating voltage step depend on the duration of inactivation. The durations (colored horizontal bars) are 100 [ms] (cyan), 200 [ms] (blue), 500 [ms] (green), 1,000 [ms] (orange), 2,000 [ms] (gray), and 5,000 [ms] (red), respectively.

Here, $\tau_{1A}$, $\tau_{A3}$, $\tau_{32}$, $\tau_{21}$, *P*_1,max_, *P*_2,max_ are kinetic parameters (Table S1), and *u* represents the release probability of presynaptic neurotransmitter, which is determined by the activation function of the presynaptic membrane potential: *V*_pre_, *V*_th_, and *V*_slp_ are the presynaptic membrane potential, the half voltage for activation of Ca^2+^ channels, and the slope of activation function of Ca^2+^ channels, respectively. This presynaptic action is similar to the previous model (Schröder et al., 2021). In addition to the presynaptic action, our model incorporated the postsynaptic action (Eq. S5 in the supplemental text at the end of the PDF). We confirmed that this synapse model could reproduce the kinetics of ribbon synapses (Fig. S2) (Grabner et al., 2016). The connection patterns and number of connections for each cell were determined based on the findings of previous studies (Jeon et al., 1998; Tsukamoto et al., 2001; Veruki and Hartveit, 2002; Margolis and Detwiler, 2007; Völgyl et al., 2009; Arai et al., 2010; Arman and Sampath, 2012; Tsukamoto and Omi, 2013, 2017; Baden et al., 2016) as summarized in Table S2. In this model, photoreceptor cells were not included, but light stimulation was incorporated as input currents to the BC model that mimicked the synaptic currents evoked by photoreceptors, i.e., a hyperpolarizing current to OFF CBCs and a depolarizing current to RBCs and ON CBCs. The source codes of the proposed models are registered to ModelDB (https://modeldb.science/2019896) and GitHub (https://github.com/ktnrktn/InnerRetina/tree/master).

For comparison with experimental results, the retinal neural circuit models were modified by setting the input currents to BCs:

- The WT circuit model: the input currents representing photoreceptor signals were applied to all BC types.
- The *Trpm1* KO circuit model: TRPM1 channels are expressed only on the dendrites of ON BCs (RBCs and ON CBCs). We hypothesized that light signals are not transmitted to downstream cells in the ON pathway due to the absence of TRPM1 function. Consistent with this hypothesis, we found that the membrane potential of RBCs is hyperpolarized in these mice (Fig. 4, A and B). Therefore, sustained hyperpolarizing currents were applied to RBCs to induce hyperpolarization, whereas no input currents were applied to ON CBCs. Input currents representing photoreceptor signals comparable to those in the WT circuit model were applied to OFF CBCs.
- The *mGluR6* KO circuit model: mGluR6 is also expressed only on the dendrites of ON BCs. Loss of mGluR6 leads to hyperpolarization of RBCs (Xu et al., 2012). Therefore, we assume that BCs in the *mGluR6* KO model behave similar to those in the *Trpm1* KO circuit model. As in the *Trpm1* KO circuit model, sustained hyperpolarizing currents were applied to RBCs to induce hyperpolarization, whereas no input currents were applied to ON CBCs. Input currents representing photoreceptor signals comparable to those in the WT circuit model were applied to OFF CBCs.
- The *rd1* circuit model: in the *rd1* mouse retina, photoreceptor degeneration leads to the loss of glutamate release from photoreceptors and the loss of TRPM1 channels in ON BC dendrites (Fig. 3 N) (Križaj et al., 2010). Based on these alterations, the hyperpolarization observed in RBCs in the *Trpm1* KO mouse retina (Fig. 4), and a previous report showing a similar state in the *rd1* mouse retina (Borowska et al., 2011), we hypothesized that RBCs are also hyperpolarized in the *rd1* mouse retina. As in the *Trpm1* KO and *mGluR6* KO circuit models, sustained hyperpolarizing currents were applied to RBCs to induce hyperpolarization. No input currents were applied to OFF and ON CBCs.

In addition, we imposed the conditions based on the experimental evidence (2) and explored the conditions where oscillatory firing was evoked in appropriate RGCs (3).

A detailed description of the computational simulation is provided in the supplemental text at the end of the PDF.

### Statistical analysis

Data were presented as the mean ± SEM, except for immunohistochemical data, which were presented as the mean ± SD. Error bars indicate SEM unless otherwise noted. For pharmacological data, statistical significance was evaluated using a two-sided paired *t* test. For immunohistochemical data, multiple comparisons were performed using one-way ANOVA with post hoc Tukey’s test. Other comparisons were made using two-sided unpaired Student’s *t* test. A P value <0.05 was considered statistically significant (n.s., not significant; *P < 0.05; **P < 0.01; ***P < 0.001).

### Online supplemental material

Fig. S1 illustrates the states of neurotransmitters of a ribbon synapse and the transition between them. Fig. S2 displays time courses of response recovery of the ribbon synapse model. Table S1 summarizes parameters values of the ribbon synapse model. Table S2 summarizes parameters of synaptic connectivity between cells and synaptic conductance, and the values of the synaptic conductance (the upper row in each cell) and the connectivity ratio (pre:post; the bottom row in each cell). Supplemental text at the end of the PDF contains a detailed description of the computational simulation used in this paper.

## Results

### Identification of αRGC subtypes in the *Trpm1* KO mouse

**Figure 1.**
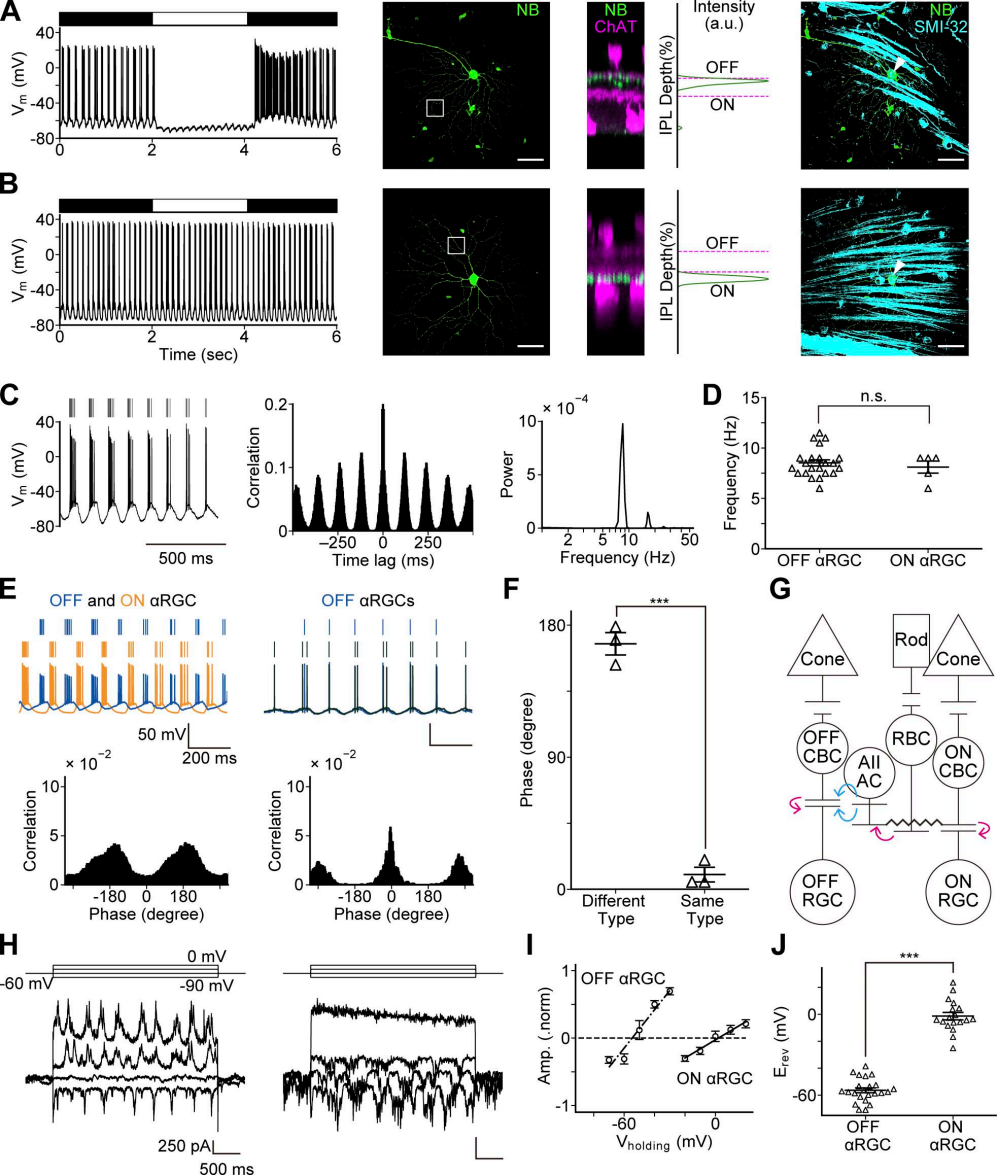
**Identification of αRGC subtypes and characterization of their oscillatory properties. (A)** Light-evoked response and morphology of an OFF αRGC. Membrane potential change to light stimulation in an OFF αRGC (left). Whole-mount view of an OFF αRGC (center). The white rectangle indicates an ROI. Side view of the stacked NB and ChAT images in the ROI. A plot of labeling intensity versus IPL depth indicates that dendritic arborization of the OFF αRGC (NB: green) is relative to the position of ChAT bands (magenta) in the ROI. The OFF αRGC dendrites stratify in the OFF sublamina. Whole-mount view of the stacked NB-stained OFF αRGC image (green) and SMI-32 images (cyan) (right). The white arrowhead indicates colocalization of the OFF αRGC soma with the SMI-32 signal. **(B)** Light response and morphology of an ON αRGC. No obvious light-evoked response was observed in an ON αRGC (left). Morphological analysis in an ON αRGC (center). The ON αRGC dendrites stratify in the ON sublamina. Whole-mount view of the stacked ON αRGC image (green) and SMI-32 images (cyan) (right). Scale bar: 50 µm. **(C)** RGC oscillations in the *Trpm1* KO mouse retina. Membrane potential trace from an OFF αRGC under the current-clamp condition (left). Spike events (top) and voltage trace (bottom). Autocorrelogram (center) is calculated from the spike events (left). The PSD (right) is derived from the autocorrelogram (center). A clear peak was detected at 8.5 Hz. **(D)** The frequency of oscillations in OFF and ON αRGCs (OFF αRGCs: 8.5 ± 0.3 Hz, *n* = 22 cells from nine mice; ON αRGCs: 8.1 ± 0.6 Hz, *n* = 5 cells from five mice; P = 0.55). **(E)** Phase relationship of oscillatory firing between αRGC pairs. Top: Membrane potential changes (bottom) and spike events (top) obtained from a pair of OFF (blue) and ON (orange) αRGCs (left), and from a pair of OFF (blue) and OFF (black) αRGCs (right). Bottom: CCRs calculated from the spike events. **(F)** Phase difference of αRGC oscillations: different-type: 167.3 ± 7.6°, *n* = 3 cell pairs from three mice; same-type: 10.0 ± 5.0°, *n* = 3 cell pairs from two mice; P = 1.89 × 10^−5^. **(G)** Schematic diagram of the rod pathway including an AII AC. Excitatory (magenta) and inhibitory (cyan) synapses, as well as gap junction (▬), are illustrated. **(H)** Synaptic currents (bottom) measured at different holding potentials (top) from an OFF αRGC (left) and an ON αRGC (right) under the whole-cell voltage-clamp condition. **(I)** Amplitude of synaptic currents at different holding potentials (OFF αRGCs: *n* = 23 cells from 19 mice; ON αRGCs: *n* = 18 cells from 16 mice). **(J)** Reversal potentials of oscillatory synaptic currents in OFF and ON αRGCs (OFF αRGCs: −56.4 ± 1.8 mV, *n* = 23 cells from 19 mice; ON αRGCs: −1.3 ± 2.8 mV, *n* = 18 cells from 16 mice; P = 8.03 × 10^−20^). Data are presented as the mean ± SEM. An unpaired *t* test was used for statistical analysis. n.s., not significant; ***P < 0.001. ROI, region of interest.

Previous MEA recordings demonstrated that RGCs of the *Trpm1* KO mouse retina generate spontaneous oscillatory firing (Takeuchi et al., 2018). To elucidate the mechanism underlying these oscillations, we performed whole-cell recordings from αRGCs (soma size >15 µm) in flat-mount retinas of *Trpm1* KO mice. It should be noted that ON BCs do not respond to light illumination due to the deletion of TRPM1 channels. Whole-cell current-clamp recordings revealed that spot illumination elicited OFF responses in some αRGCs (Fig. 1 A, left), while others showed no light responses (Fig. 1 B, left). To confirm that the light-responsive and nonresponsive cells corresponded to OFF and ON αRGCs, respectively, we examined the morphology of RGCs filled with NB through a recording pipette. RGC subtypes were determined by their dendritic stratification relative to the ChAT bands (dendritic stratification of OFF and ON starburst ACs) in the IPL. The OFF- and ON-ChAT bands in the IPL were defined as 0% and 100%, respectively. Consistent with their light responses, RGCs with light-Off responses had dendrites that stratified in the OFF sublamina (Fig. 1 A, center), whereas those without responses stratified their dendrites in the ON sublamina (Fig. 1 B, center). Furthermore, immunohistochemical staining for SMI-32, an αRGC marker, confirmed that the recorded RGCs were indeed αRGCs (Fig. 1, A and B, right, arrowheads). Therefore, in the following experiments, the subtype of recorded RGCs was determined based on this postrecording morphological analysis.

### Fundamental frequency of oscillations in OFF αRGCs was similar to that in ON αRGCs

To examine the properties of spontaneous oscillatory discharges in αRGCs, cells were whole-cell current-clamped using a patch pipette filled with K^+^-based solution. Spike discharges were evoked when the membrane potential fluctuations reached a threshold (Fig. 1 C, left). An autocorrelogram was calculated from the timing of these spike discharges (Fig. 1 C, center). The PSD derived from the autocorrelogram revealed that the fundamental frequency of oscillations was ∼8–9 Hz (Fig. 1 C, right) in both OFF and ON αRGCs (Fig. 1 D). These results may be explained either by common inputs from a presynaptic oscillator or by their own similar intrinsic oscillators.

### Phase of oscillations was anti-phase in a pair of different αRGC types

To examine the phase relationship of oscillations, we performed simultaneous whole-cell current-clamp recordings from pairs of αRGCs. CCRs were calculated from their spike discharges, and the time lag was converted to phase using the fundamental frequency of oscillations in each RGC pair (Fig. 1 E). The phase of oscillations was anti-phase between a pair of OFF and ON αRGCs, whereas it was in-phase between a pair of the same αRGC type. This difference in phase relationship was statistically significant (Fig. 1 F). These findings are consistent with those previously reported in *rd1* and *Nyx*^*nob*^ mouse retinas (Margolis et al., 2014; Winkelman et al., 2019), and suggest the idea that oscillations in OFF and ON αRGCs are driven by synaptic inputs from common presynaptic oscillators.

### OFF and ON αRGC oscillations were generated by different synaptic inputs

To determine whether αRGC oscillations were driven by synaptic inputs rather than intrinsic voltage-dependent membrane properties, αRGCs were whole-cell voltage-clamped using a patch pipette filled with Cs^+^-based solution to suppress K^+^ channels. Under these conditions, oscillatory membrane currents were evident, indicating that the oscillations were indeed driven by synaptic inputs rather than intrinsic properties of αRGCs. The mean frequency of these synaptic current oscillations was 3.1 ± 0.2 Hz (*n* = 41 cells from 28 mice), which was significantly lower than that observed during current-clamp recordings with K^+^-based pipette solution (8.5 ± 0.3 Hz, *n* = 27 cells from 9 mice) (P = 4.64 × 10^−26^, unpaired Student’s *t* test). This frequency difference might be attributable to the blockade of I_h_ channels (Kaneko and Tachibana, 1985; Müller et al., 2003; Trenholm et al., 2012) and/or M-type K^+^ channels (Cembrowski et al., 2012; Choi et al., 2014) by Cs^+^ extruded from a patch pipette before establishing the cell-attached configuration.

To further characterize the oscillatory synaptic inputs, synaptic currents were recorded at various holding potentials in both OFF and ON αRGCs (Fig. 1 H). The amplitudes of these synaptic currents (see Materials and methods) were plotted against their corresponding holding potentials (Fig. 1 I). The reversal potential of the synaptic currents differed significantly between OFF and ON αRGCs (Fig. 1 J). These results suggest that oscillations in OFF αRGCs are driven by inhibitory (glycinergic and/or GABAergic) synaptic inputs, while those in ON αRGCs are driven by excitatory (glutamatergic) synaptic inputs. The observed differences in synaptic inputs are consistent with the known retinal circuitry involving AII ACs. AII ACs form gap junctions with both ON CBCs and other AII ACs and make glycinergic synapses onto OFF CBCs and some OFF RGCs (Fig. 1 G) (Feigenspan et al., 2001; Murphy and Rieke, 2006; Murohy and Rieke, 2008; Münch et al., 2009; Tsukamoto and Omi, 2017). Therefore, it is likely that OFF αRGCs receive direct glycinergic inputs from AII ACs, whereas ON αRGCs may receive glutamatergic inputs from ON CBCs, which are connected to AII ACs through gap junctions (Fig. 1 G).

### Blockade of AII AC pathways eliminates oscillatory synaptic currents in αRGCs

**Figure 2.**
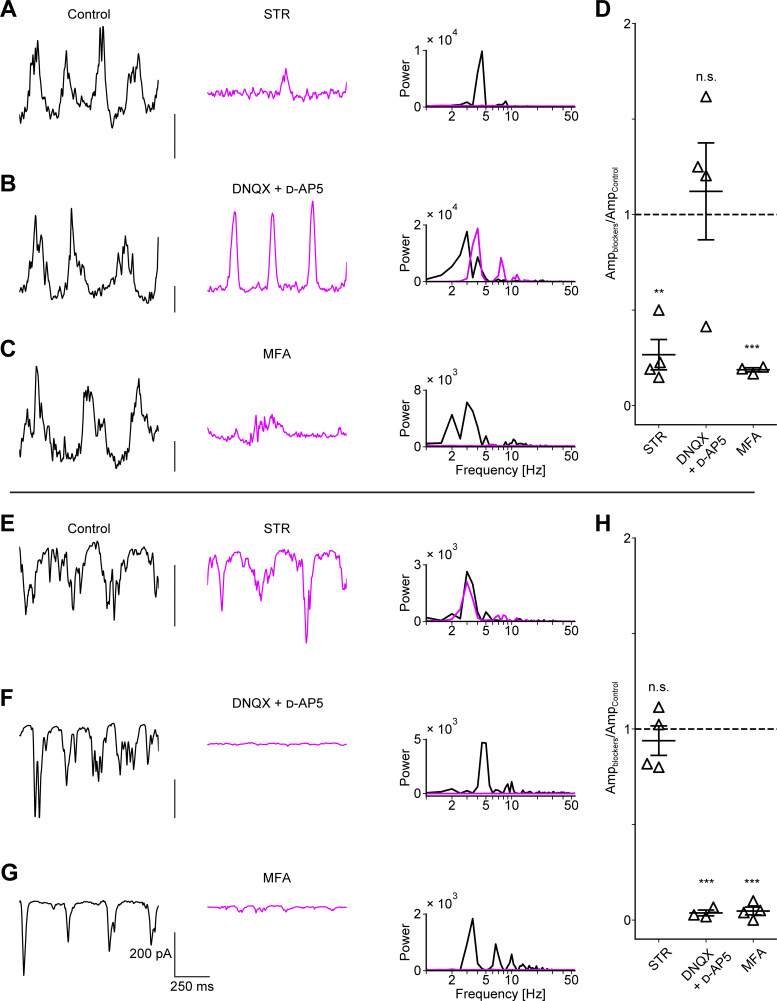
**Oscillatory synaptic currents are eliminated by blockers, which affect the pathways involving AII AC. (A–C)** Effects of synaptic blockers on IPSCs in OFF αRGCs. IPSCs recorded from OFF αRGCs in the control condition (left). Effects of blockers on IPSCs (center). PSDs in the control condition (black) and blocker conditions (magenta) (right). **(D)** Ratios of IPSC amplitudes in the presence of blockers to those under control conditions in OFF αRGCs (STR: 0.27 ± 0.08, *n* = 4 cells from three mice; P = 2.69 × 10^−3^, DNQX + d-AP5: 1.12 ± 0.25, *n* = 4 cells from four mice; P = 0.66, MFA: 0.19 ± 0.01, *n* = 3 cells from three mice; P = 1.71 × 10^−4^). **(E–G)** Effects of synaptic blockers on EPSCs in ON αRGCs. Layouts and analyses are the same as in A–C. **(H)** Ratios of EPSC amplitudes in the presence of blockers to those under control conditions in ON αRGCs (STR: 0.94 ± 0.08, *n* = 4 cells from three mice; P = 0.49, DNQX + d-AP5: 0.04 ± 0.01, *n* = 3 cells from three mice; P = 2.42 × 10^−4^, MFA: 0.05 ± 0.02, *n* = 4 cells from four mice; P = 2.22 × 10^−5^). Data are presented as the mean ± SEM. A paired *t* test was used for statistical analysis. n.s., not significant; **P < 0.01; ***P < 0.001. STR: strychnine.

We conducted pharmacological experiments to investigate the synaptic pathways responsible for transmitting oscillatory signals to αRGCs. OFF αRGCs were voltage-clamped at 0 mV to isolate oscillatory inhibitory postsynaptic currents (IPSCs). Bath application of strychnine (10 µM), a glycine receptor antagonist, significantly reduced both the amplitude of IPSCs and the peak power in the PSD (Fig. 2, A and D). In contrast, application of iGluR antagonists (a mixture of 100 µM DNQX and 50 µM d-AP5) affected neither the amplitude of IPSCs nor the peak power in the PSD (Fig. 2, B and D). This result suggests that oscillatory IPSCs in OFF αRGCs may be driven directly by AII ACs rather than via other ACs through iGluR-mediated synapses. Application of MFA (100 µM), a nonspecific gap junction blocker, also significantly reduced both the amplitude of IPSCs and the peak power in the PSD (Fig. 2, C and D). Based on the circuit diagram shown in Fig. 1 G, the effects of glycine receptor and iGluR antagonists were consistent with expectations. However, the effect of the gap junction blocker was unexpected. It is possible that gap junction blockade hyperpolarizes AII ACs and abolished oscillatory activity in AII ACs themselves (see Fig. 7 C) (Trenholm et al., 2012; Choi et al., 2014).

ON αRGCs were voltage-clamped at −60 mV to record oscillatory excitatory postsynaptic currents (EPSCs). Application of strychnine (10 µM) affected neither the amplitude of EPSCs nor the peak power in the PSD (Fig. 2, E and H). In contrast, application of iGluR antagonists (a mixture of 100 µM DNQX and 50 µM d-AP5) abolished both the oscillatory EPSCs and the peak power in the PSD (Fig. 2, F and H). MFA (100 µM) also significantly reduced the amplitude of EPSCs and the peak power in the PSD (Fig. 2, G and H). These findings are consistent with the synaptic connections outlined in Fig. 1 G.

### Position and size of RBC axon terminals

**Figure 3.**
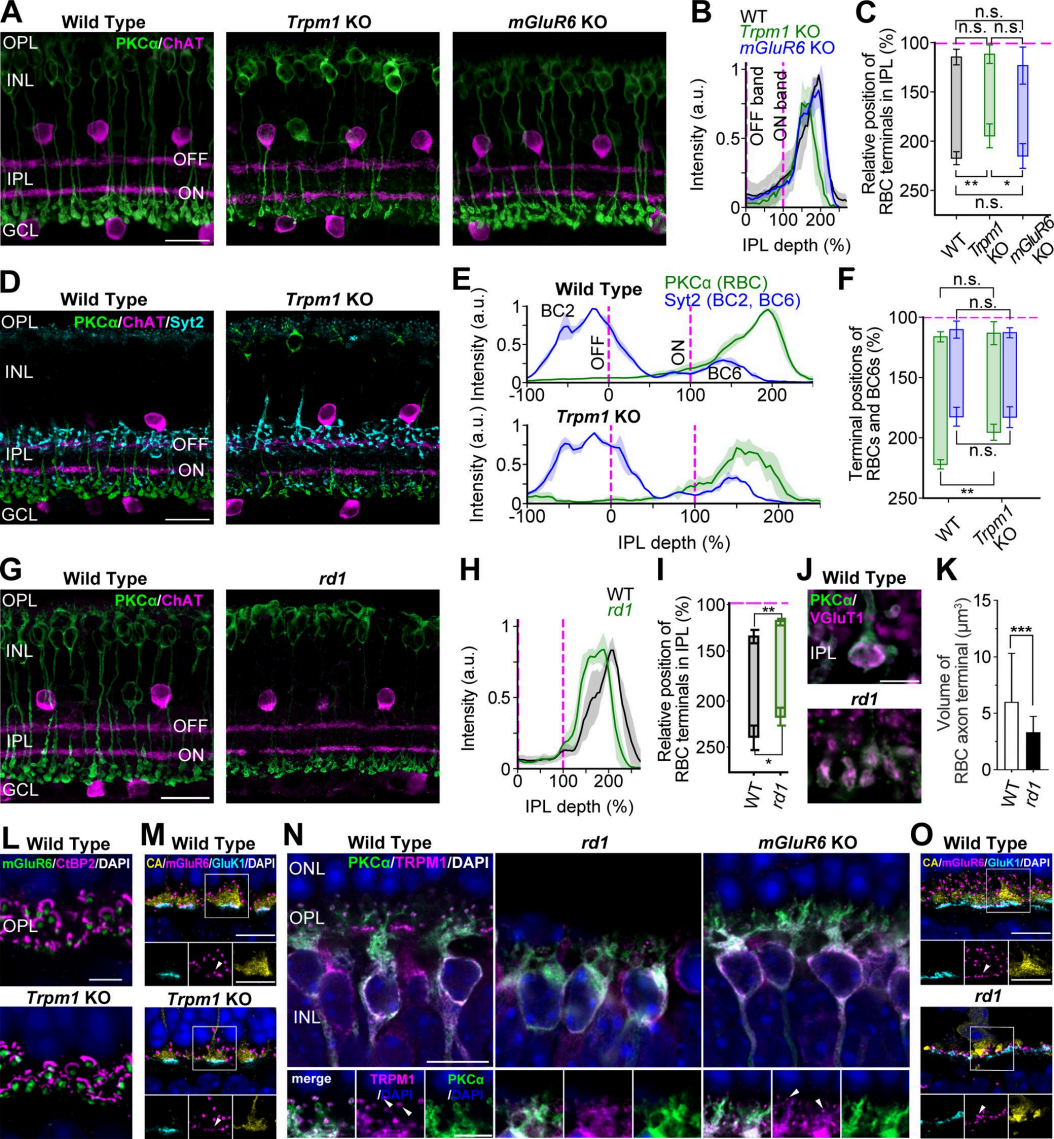
**Common morphological features and marker expression in the *Trpm1* KO and *rd1* mouse retinas. (A–C)** Distribution of RBC terminals in the WT, *Trpm1* KO, and *mGluR6* KO mouse retinas. **(A)** RBCs and SACs were immunolabeled with PKCα (green) and ChAT (magenta: IPL sublaminar maker), respectively. Scale bar: 20 µm. **(B)** Intensity profiles of RBC labeling from A relative to the location of the ChAT bands (magenta dotted lines). Solid lines and shaded areas represent the mean ± SD, respectively. **(C)** Ending point of RBC terminal distribution in the IPL was significantly shifted toward the ON-ChAT band (magenta dotted lines) in the *Trpm1* KO mouse retinas compared with the WT and *mGluR6* KO mouse retinas (starting point: WT: 114.6 ± 7.9%, *n* = 6 sections from six mice; *Trpm1* KO: 111.8 ± 9.3%, *n* = 5 sections from five mice; *mGluR6* KO: 123.3 ± 18.8%, *n* = 3 sections from three mice; P = 0.24, ending point: WT: 217.3 ± 6.6%, *n* = 6 sections from six mice; *Trpm1* KO: 194.6 ± 12.1%, *n* = 6 sections from six mice; *mGluR6* KO: 215.1 ± 12.5%, *n* = 3 sections from three mice; P = 0.004). **(D–F)** Distribution of RBC and type 6 BC terminals in the WT and *Trpm1* KO mouse retinas. **(D)** RBCs, type 2 and 6 BCs, and SACs were immunolabeled with PKCα (green), Syt2 (cyan), and ChAT (magenta), respectively. Scale bar: 20 µm. **(E)** Intensity profiles of PKCα and Syt2 labeling from D relative to the location of the ChAT bands (magenta dotted lines). **(F)** The ending point of RBC terminal distribution (GCL side) was significantly closer to the ON-ChAT band (magenta dotted lines) in the *Trpm1* KO mouse retinas compared with the WT mouse retinas (starting point: WT: 116.3 ± 4.2%, *n* = 3 sections from three mice; *Trpm1* KO: 113.3 ± 9.5%, *n* = 3 sections from three mice; P = 0.65, ending point: WT: 222.1 ± 3.8%, *n* = 3 sections from three mice; *Trpm1* KO: 195.4 ± 6.7%, *n* = 3 sections from three mice; P = 0.004, unpaired *t* test). In contrast, the distribution of type 6 BC terminals was similar between WT and *Trpm1* KO mouse retinas (starting point: WT: 110.3 ± 7.1%, *n* = 3 sections from three mice; *Trpm1* KO: 112.9 ± 4.1%, *n* = 3 sections from three mice; P = 0.62, ending point: WT: 182.5 ± 7.8%, *n* = 3 sections from three mice; *Trpm1* KO: 182.9 ± 8.6%, *n* = 3 sections from three mice; P = 0.96, unpaired *t* test). **(G–K)** Altered distribution and reduced volume of RBC terminals in the *rd1* mouse retinas. **(G–I)** Distribution of RBC terminals was shifted toward the INL in the *rd1* mouse retinas. Similar analyses are shown in A–C (starting point: WT: 133.7 ± 7.3%, *n* = 5 sections from five mice; *rd1*: 118.5 ± 3.4%, *n* = 5 sections from five mice; P = 0.003, ending point: WT: 238.9 ± 13.2%, *n* = 5 sections from five mice; *rd1*: 217.7 ± 9.1%, *n* = 5 sections from five mice; P = 0.02). **(J and K)** Volume of RBC terminals in the *rd1* mouse retinas was smaller than that in the WT mouse retinas. **(J)** Synaptic terminals immunolabeled with PKCα (green) and VGluT1 (magenta) in the WT (top) and *rd1* (bottom) mouse retinas. Scale bar: 5 µm. **(K)** RBC terminal volumes were significantly smaller in the *rd1* mouse retinas than in the WT mouse retinas (WT: 5.7 ± 5.7 µm^3^, *n* = 107 terminals, 3 retinas, 3 mice; *rd1*: 3.4 ± 2.7 µm^3^, *n* = 86 terminals, 3 retinas, 3 mice; P = 7.00 × 10^−4^). **(L–O)** Marker expression in the OPL. **(L)** OPL localization of mGluR6 (green), CtBP2 (magenta), and DAPI (blue) was similar between WT (top) and *Trpm1* KO (bottom) mouse retinas. Scale bar: 5 µm. **(M)** Cone pedicles remained intact in the *Trpm1* KO mouse retinas. Cone arrestin (yellow), mGluR6 (magenta), GluK1 (cyan), and DAPI (blue). Scale bar: 5 µm. WT (top) and the *Trpm1* KO (bottom) mouse retinas. **(N)** TRPM1 puncta were moderately reduced in the *mGluR6* KO mouse retinas and severely diminished in the *rd1* mouse retinas. RBC dendrites showed intact structure in the *mGluR6* KO mouse retina but retracted in the *rd1* mouse retinas. WT (left), *rd1* (center), *mGluR6* KO (right) mouse retinas. Scale bar: 10 µm. Insets show an enlarged view of individual RBC dendritic trees. Scale bar: 5 µm. **(O)** Cone pedicles showed degeneration in the *rd1* mouse retinas. Cone arrestin (yellow), mGluR6 (magenta), GluK1 (cyan), and DAPI (blue). Scale bar: 5 µm. WT (top) and *rd1* (bottom) mouse retinas. Data are presented as the mean ± SD. Error bars indicate SD. n.s., not significant; *P < 0.05; **P < 0.01; ***P < 0.001. SACs, starburst ACs.

Previous studies have shown that both *Trpm1* KO and *rd1* mouse retinas exhibit prominent spontaneous oscillations in RGCs, whereas the *mGluR6* KO mouse retina shows infrequent or absent spontaneous oscillations (Margolis et al., 2008; Margolis et al., 2014; Stasheff, 2008; Borowska et al., 2011; Menzler and Zeck, 2011; Yee et al., 2012; Choi et al., 2014; Poria and Dhingra, 2015; Takeuchi et al., 2018; Hasan et al., 2020). In the WT mouse retinas, RBCs, which express both TRPM1 and mGluR6 on their dendritic membranes, provide glutamatergic output to AII ACs. Thus, it is interesting to examine whether the morphological features of RBC differ among these mouse retinas. Firstly, we focused on the position and size of RBC axon terminals. We examined the distribution of RBC terminals relative to ChAT bands in the IPL in each mouse retina. The starting point of terminal distribution in the distal IPL did not significantly differ among WT, *Trpm1* KO, and *mGluR6* KO mouse retinas (Fig. 3, A–C). However, the ending point of terminal distribution in the proximal IPL was significantly closer to the ON-ChAT band (100%) in the *Trpm1* KO mouse retinas compared with both WT and *mGluR6* KO mouse retinas (Fig. 3, A–C).

To determine whether this change in terminal distribution is specific to RBCs or common among ON BCs, we compared the distribution of type 6 BC terminals between WT and *Trpm1* KO mouse retinas. No significant difference was found in the terminal distribution of type 6 BCs between the two genotypes. In contrast, RBC terminals in the *Trpm1* KO mouse retinas showed a significantly shorter span, with their ending point located closer to the ON-ChAT band than in the WT mouse retinas (Fig. 3, D–F). These results suggest that the altered distribution of RBC terminals in the *Trpm1* KO mouse retinas is cell type–specific and not simply due to a general change in the thickness of the ON sublamina. In the *rd1* mouse retinas, the RBC terminal distribution was shifted toward the INL side (Fig. 3, G–I).

We previously reported that the volume of individual RBC axon terminals is reduced in the *Trpm1* KO mouse retinas relative to the *mGluR6* KO and WT mouse retinas, which are indistinguishable (Takeuchi et al., 2018). In the *rd1* mouse retina, a similar reduction in RBC axon terminal volume was observed (Fig. 3, J and K) (see also Strettoi et al., 2002, 2003; Chua et al., 2009). Together, these results demonstrate that prominent alterations in RBC axon terminals are present in both *Trpm1* KO and *rd1* mouse retinas, but not in the *mGluR6* KO mouse retinas. It is tempting to speculate that these morphological alterations are associated with a reduction in signaling from RBCs to AII ACs in both *Trpm1* KO and *rd1* mouse retinas.

### Marker expression in the outer plexiform layer

In the outer plexiform layer (OPL), knocking out *Trpm1* affected neither the intensity of mGluR6 puncta nor their apposition to the presynaptic rod ribbon marker rib eye (CtBP2) (Fig. 3 L), consistent with previous reports (Koike et al., 2009; Morgans et al., 2009). Similarly, knocking out *Trpm1* did not alter the colocalization of mGluR6 with GluK1 (a kainate receptor subunit: OFF CBC marker) and arrestin at cone terminals (Fig. 3 M). In contrast, the localization of TRPM1 in the OPL differed among WT and three models (*Trpm1* KO, *mGluR6* KO, and *rd1*) (Fig. 3 N). In the *mGluR6* KO mouse retinas, TRPM1 puncta were present but displayed slightly reduced intensity compared with the WT mouse retinas. In the *rd1* mouse retinas, TRPM1 puncta on RBC dendrites were markedly reduced (Fig. 3 N). This finding was consistent with the loss of rod spherules, although some cone pedicles were preserved (Fig. 3 O). The *Trpm1* KO mouse retina lacks puncta by definition (Koike et al., 2009; Morgans et al., 2009). These results suggest a three-way association with RGC oscillations: the extent of TRPM1 loss at dendrites, the altered distribution of RBC terminals, and the shrinkage of RBC axon terminals.

### RBC membrane potential was hyperpolarized in the *Trpm1* KO mouse retina

**Figure 4.**
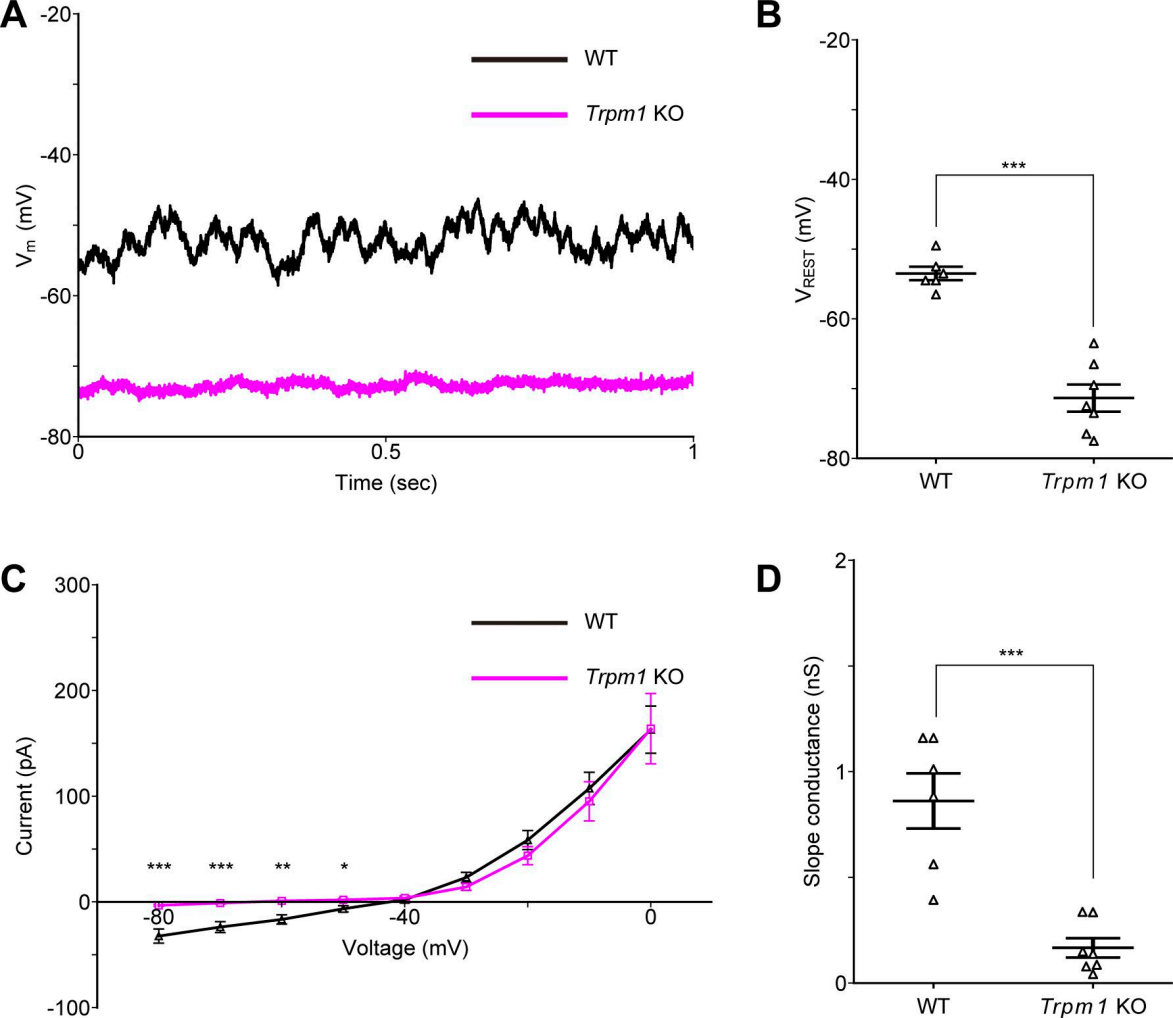
**Resting membrane potential of RBCs in WT and *Trpm1* KO mouse retinas. (A)** RBC membrane potential traces recorded from the WT (black) and *Trpm1* KO (magenta) mouse retinas. **(B)** Resting membrane potential of RBCs in the WT and *Trpm1* KO mouse retinas (WT: −53.5 ± 1.0 mV, *n* = 6 cells from five mice; *Trpm1* KO: −71.4 ± 1.9 mV, *n* = 7 cells from six mice; P = 8.4 × 10^−6^). **(C)** Averaged I–V curves of RBCs in WT (triangle, black, *n* = 6 cells from five mice) and *Trpm1* KO (square, magenta, *n* = 7 cells from six mice) mouse retinas. −80 mV: P = 0.0007; −70 mV: P = 0.0007; −60 mV: P = 0.001; −50 mV: P = 0.02. **(D)** Slope conductance of RBCs in the WT and *Trpm1* KO mouse retinas (WT: 0.86 ± 0.13 nS, *n* = 6 cells from five mice; *Trpm1* KO: 0.17 ± 0.05 nS, *n* = 7 cells from six mice; P = 0.0002). Data are presented as the mean ± SEM. An unpaired *t* test was used for statistical analysis. n.s., not significant; *P < 0.01; **P < 0.01; ***P < 0.001.

Our immunohistochemical analyses revealed morphological alterations of RBCs in mouse retinas that exhibit RGC oscillations. Based on these findings, we next investigated potential functional changes, specifically focusing on the synaptic input from RBCs to AII ACs. It seems likely that the neurotransmitter release from neurons, including RBCs, depends on their membrane potential (Snellman et al., 2009; Graydon et al., 2018). Thus, using retinal slice preparation, we measured the resting membrane potential of RBCs. The resting membrane potential of RBCs in the *Trpm1* KO mouse retinas was significantly hyperpolarized compared with those in the WT mouse retinas (Fig. 4, A and B). This may result from the loss of the TRPM1 channels. To test this possibility, we analyzed the I–V relationship of RBCs. We found that the slope conductance, calculated from the linear range of the I–V curve (Fig. 4 C) near the resting membrane potential, was significantly lower in the *Trpm1* KO mouse retinas than in the WT mouse retinas (Fig. 4 D). Therefore, the hyperpolarization of RBCs in the *Trpm1* KO mouse retinas may cause reduced glutamate release from RBCs to AII ACs.

### Reproduction of oscillations based on common morphological alterations in the *Trpm1* KO and *rd1* mouse retinas

We constructed retinal circuit models to examine whether reduced synaptic input from RBCs to AII ACs can generate oscillatory firing in RGCs. First, we developed a normal retinal circuit model (the WT circuit model) as described in Materials and methods (see also supplemental text at the end of the PDF). Second, we modified the input currents to BCs in each mouse circuit model to reproduce the light-evoked responses, as detailed in Materials and methods (see also supplemental text at the end of the PDF). Third, to reflect the shrinkage of RBC axon terminals observed in the *Trpm1* KO and *rd1* mouse retinas (Fig. 3) (Takeuchi et al., 2018), we reduced the synaptic conductance from RBCs to AII ACs in those circuit models.

In the mammalian retina, RBCs provide excitatory drive to AII ACs, which in turn are electrically coupled to ON CBCs via gap junctions (Fig. 1 G). In the *Trpm1* KO and *rd1* mouse retinas, the loss of TRPM1 channels induces severe defects in RBCs, including its absence or a marked reduction of the channel at their dendritic tips (Koike et al., 2009) (Fig. 3 N), altered axon terminal morphology (Takeuchi et al., 2018) (Fig. 3), and a hyperpolarized resting membrane potential (Fig. 4) (Borowska et al., 2011). Collectively, these deficits are expected to reduce the excitatory drive to AII ACs, consequently leading to a hyperpolarizing shift in the resting membrane potential of the coupled ON CBCs. Furthermore, the loss of the light-evoked responses in ON CBCs in these mouse retinas may also contribute to their hyperpolarization (Lagali et al., 2008; Koike et al., 2009). Since ON CBCs express T-type Ca^2+^ channels (de la Villa et al., 1998; Pan, 2000; Zhang et al., 2022), we hypothesized that these channels might allow glutamate release even at hyperpolarized membrane potentials. This condition was reproduced in those specific models by lowering the half-activation voltage of Ca^2+^ channels (see *V*_*th*_ in Eq. 5 in Materials and methods and additional details in the supplemental text at the end of the PDF) in the ON CBC terminals.

**Figure 5.**
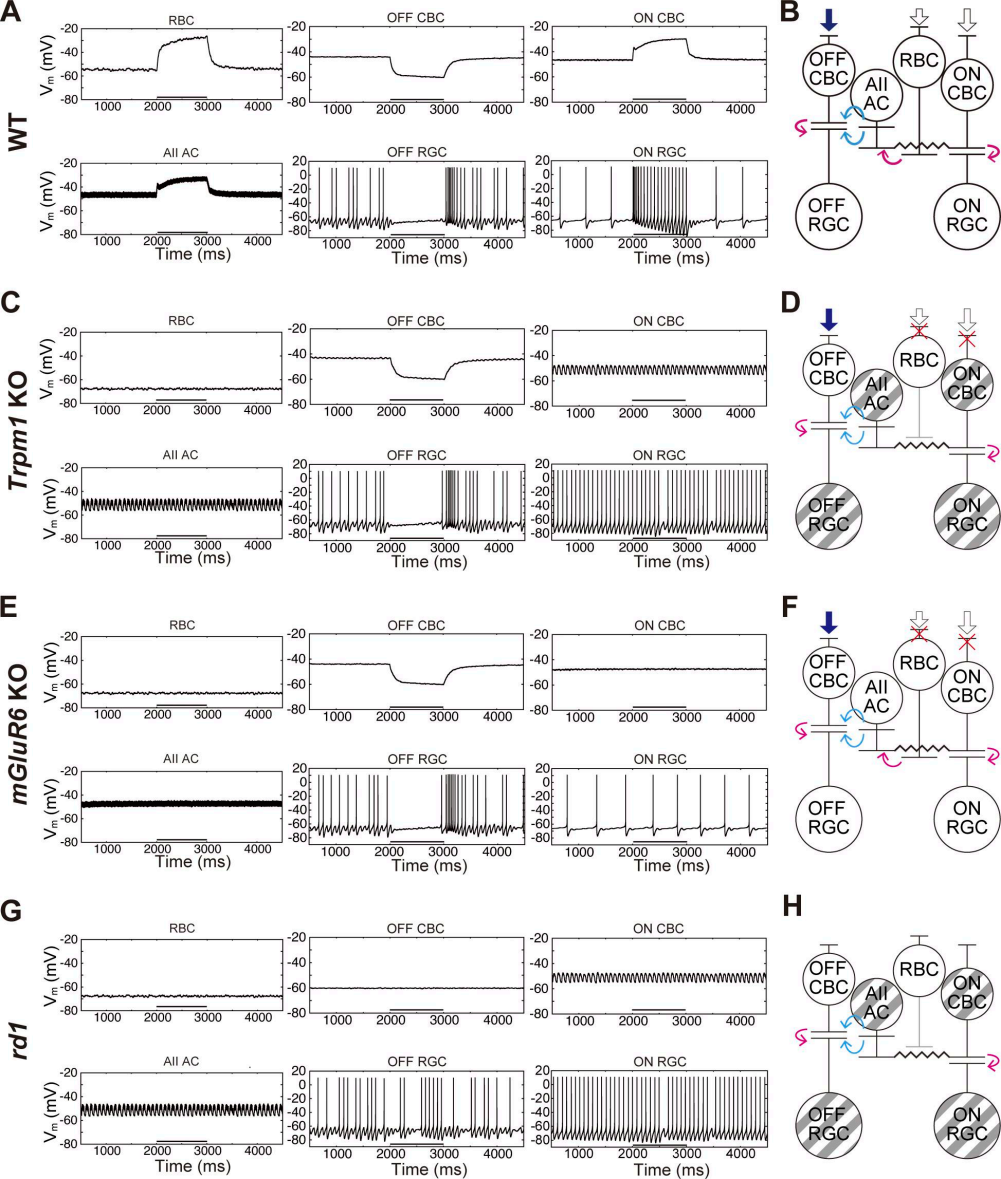
**Numerical simulation of retinal neural circuit models. (A)** Simulated membrane potential changes in RBCs, OFF CBCs, ON CBCs, AII ACs, OFF RGCs, and ON RGCs in the WT circuit model. Light stimulation (black bar: bottom) hyperpolarizes OFF CBCs, while it depolarizes RBCs, ON CBCs, and AII ACs. The firing rate increases at light offset in OFF RGCs and at light onset in ON RGCs. **(B)** Schematic diagram of the WT circuit model. Filled and open arrows to BCs indicate hyperpolarizing and depolarizing current inputs, respectively. Excitatory (magenta) and inhibitory (cyan) synapses, as well as gap junctions (▬), are illustrated. **(C)** Simulated membrane potential changes in the *Trpm1* KO circuit model. OFF CBCs and OFF RGCs respond to light stimulation. RBCs, ON CBCs, AII ACs, and ON RGCs show no light responses due to the loss of TRPM1. ON CBCs and AII ACs exhibit membrane oscillations. Both OFF and ON RGCs show spontaneous oscillatory firing. **(D)** Schematic diagram of the *Trpm1* KO circuit model. Due to the loss of TRPM1, RBCs and ON CBCs do not respond to light stimulation. The cells with oscillations are shown by striped patterns. **(E)** Simulated membrane potential changes in the *mGluR6* KO circuit model. OFF CBCs and OFF RGCs respond to light stimulation. RBCs, ON CBCs, AII ACs, and ON RGCs show no light responses due to the loss of mGluR6. ON CBCs and AII ACs do not exhibit marked membrane oscillations. **(F)** Schematic diagram of the *mGluR6* KO circuit model. Due to the loss of mGluR6, RBCs and ON CBCs do not respond to light stimulation. **(G)** Simulated membrane potential changes in the *rd1* circuit model. RBCs, ON CBCs, OFF CBCs, AII ACs, OFF RGCs, and ON RGCs show no light responses due to photoreceptor degeneration. ON CBCs and AII ACs exhibit membrane oscillations, similar to those observed in the *Trpm1* KO circuit model (C). OFF and ON RGCs show spontaneous oscillatory firing. **(H)** Schematic diagram of the *rd1* circuit model. Due to photoreceptor degeneration, BCs do not receive synaptic currents from photoreceptors. The cells with oscillations are shown by striped patterns.

Using these retinal circuit models, we simulated the membrane potential changes of each cell. In the WT circuit model, all cell types (RBCs, OFF and ON CBCs, AII ACs, and OFF and ON RGCs) responded to light stimulation, and no membrane oscillations were observed (Fig. 5, A and B).

In the *Trpm1* KO circuit model (Fig. 5, C and D), RBCs were hyperpolarized and showed no ON response. OFF CBCs exhibited weak membrane oscillations and a hyperpolarizing light response. ON CBCs and AII ACs were hyperpolarized due to reduced inputs from RBCs to AII ACs and displayed membrane oscillations without ON responses. Both OFF and ON RGCs showed oscillatory firing, but only OFF RGCs responded to light stimulation.

In the *mGluR6* KO circuit model (Fig. 5, E and F), RBCs were hyperpolarized and lacked ON responses. OFF CBCs exhibited OFF responses to light stimulation but no obvious membrane oscillations. ON CBCs and AII ACs showed neither ON responses nor membrane oscillations. OFF and ON RGCs exhibited spontaneous firing, but only OFF RGCs responded to light stimulation. Such firing is unlikely to represent true oscillations, as they were not synchronized with fast membrane fluctuations of AII ACs. Rather, they may reflect intrinsic membrane properties of RGCs, which were not observed in our physiological recordings under the whole-cell voltage-clamp condition.

Notably, the circuit model reproduced RGC oscillations only in the *Trpm1* KO circuit model but not in the *mGluR6* KO circuit model. The critical difference between *Trpm1* KO and *mGluR6* KO circuit models is in the synaptic conductance from RBCs to AII ACs. In the *Trpm1* KO circuit model, this conductance was reduced to 0 due to axon terminal shrinkage, whereas it remained normal in the *mGluR6* KO circuit model, comparable to the WT levels. These findings suggest that reducing RBC-to-AII AC synaptic conductance may be essential for inducing membrane oscillations in AII ACs. Supporting this conclusion, the *rd1* circuit model, which also included reduced synaptic conductance from RBCs to AII ACs, reproduced membrane oscillations (Fig. 5, G and H).

### Reduced RBC synaptic input and ON CBC hyperpolarization drive AII AC oscillations

**Figure 6.**
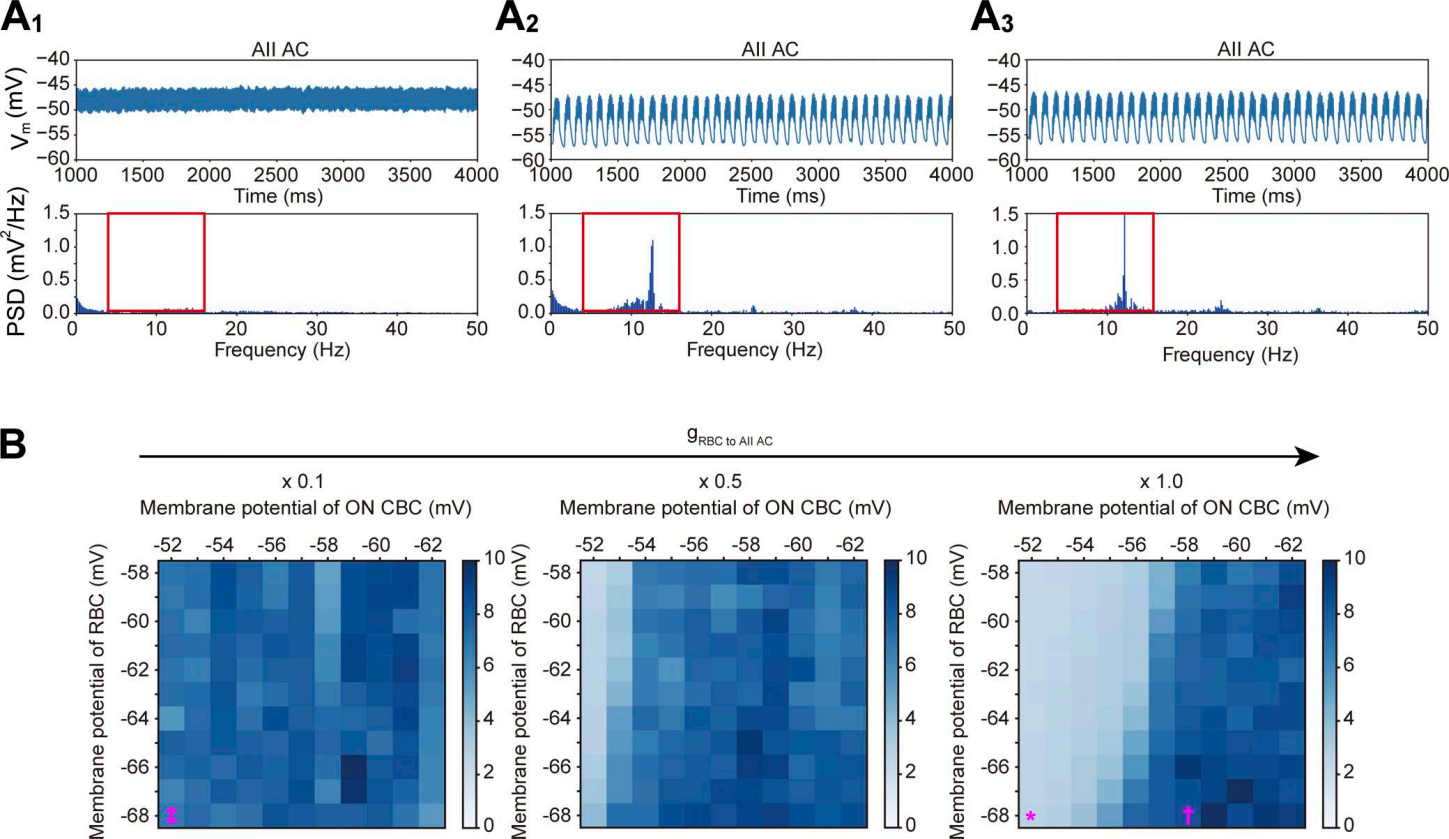
**Dependence of AII AC oscillations on RBC-to-AII AC synaptic conductance and ON CBC membrane potential. (A)** Representative simulation results under three different conditions (A_1_–A_3_). In each panel, the top trace shows the simulated AII AC membrane potential, and the bottom plot shows the corresponding PSD. The red square in PSD indicates the 5–15 Hz ROI used for analysis. **(A**_**1**_**)** Simulation with the membrane potential of −68 mV for RBC and −52 mV for ON CBC, and a g_RBC to AII AC_ of 1.0 (comparable to WT). **(A**_**2**_**)** Simulation with the ON CBC membrane potential hyperpolarized to −58 mV. Other parameters are the same as in A_1_. **(A**_**3**_**)** Simulation with g_RBC to AIIAC_ reduced to 0.1. Other parameters are the same as in A_1_. **(B)** Summary of the relationship between the strength of AII AC oscillations and the membrane potential of RBC and ON CBC. The synaptic conductance from RBC to AII AC was changed. The symbols indicate the simulated conditions shown in A_1_ (*), A_2_ (†), and A_3_ (‡). Color intensity represents the integrated value of the PSD calculated from the ROI.

To evaluate how the membrane potential of RBC and ON CBC influences AII AC oscillations, we systematically manipulated parameters of the *Trpm1* KO circuit model. Although our original *Trpm1* KO circuit model exhibited membrane oscillations in AII ACs (Fig. 5 C), these oscillations disappeared when the synaptic conductance from RBC to AII AC (g_RBC to AII AC_) was set to 1.0 (a value comparable to the WT circuit model) and the membrane potential of RBC and ON CBC was set to −68 and −52 mV, respectively (Fig. 6 A_1_). In contrast, marked membrane oscillations were observed in AII ACs when the ON CBC membrane potential was hyperpolarized to −58 mV, while the RBC membrane potential and g_RBC to AII AC_ were kept at −68 mV and 1.0, respectively (Fig. 6 A_2_). Furthermore, marked membrane oscillations were also observed in AII ACs when g_RBC to AII AC_ was reduced to 0.1, while the membrane potential of RBC and ON CBC remained the same (RBC: −68 mV, ON CBC: −52 mV; Fig. 6 A_3_). A summary of the effects of the membrane potential of RBC and ON CBC on the AII AC activity is shown in Fig. 6 B. These simulations suggest that the membrane potential of ON CBC may play a more critical role in generating AII AC oscillations than that of RBC (Fig. 6, A_1_ and A_2_). Moreover, reducing the synaptic conductance from RBC to AII AC also had a significant impact on generating AII AC oscillations (Fig. 6, A_1_ and A_3_). Taken together, these results suggest that both reduction of the synaptic conductance from RBC to AII AC and hyperpolarization of ON CBC are key factors for generating AII AC oscillations.

### Simulation of blocker effects on RGC firing

**Figure 7.**
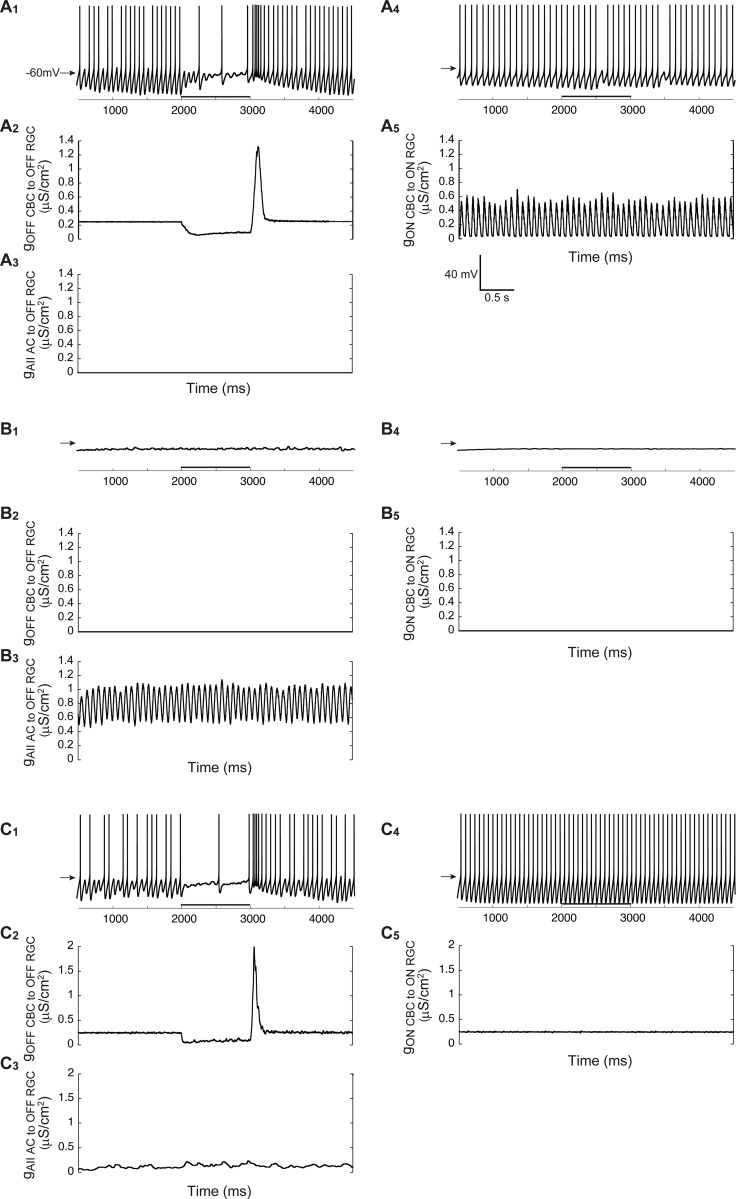
**Simulation of pharmacological blockade in the *Trpm1* KO circuit model. (A**_**1**_ **–A**_**5**_ **)** Simulated blockade of glycinergic synaptic transmission. Each panel shows the membrane potential change in OFF RGC (A_1_), synaptic conductance from OFF CBC to OFF RGC (A_2_), synaptic conductance from AII AC to OFF RGC (A_3_), membrane potential change in ON RGC (A_4_), and synaptic conductance from ON CBC to ON RGC (A_5_). Horizontal bars indicate light stimulation. **(B_1_–B_5_)** Simulated blockade of glutamatergic synaptic transmission. Panel arrangement is as in A. **(C_1_–C_5_)** Simulated blockade of gap junctions. Panel arrangement is as A and B.

Using the *Trpm1* KO circuit model, we simulated the effects of pharmacological blockers by selectively modifying the relevant synaptic or gap junctional conductance. First, to mimic the effect of a glycine receptor antagonist, we set g_glycine_ to 0 nS. This manipulation eliminated inhibitory input from AII AC to OFF RGC (Fig. 7 A_3_), resulting in tonic excitatory input from OFF CBC to OFF RGC (Fig. 7 A_2_). Consequently, this tonic excitatory input but not periodic input evoked continuous firing in OFF RGC during the epoch without light stimulation (Fig. 7 A_1_). OFF RGCs themselves or other GABAergic ACs may contribute to OFF RGC continuous firing. In response to light stimulation, OFF CBC reduced the excitatory input to OFF RGC (Fig. 7 A_2_), resulting in an OFF response (Fig. 7 A_1_). Notably, this parameter change did not affect the ON pathway (Fig. 7, A_4_ and A_5_).

Second, the effects of iGluR antagonist were mimicked by setting g_iGluR_ to 0 nS. Under this condition, oscillations in both OFF and ON RGCs disappeared (Fig. 7, B_1_ and B_4_). Excitatory inputs were eliminated from OFF CBC to OFF RGC (Fig. 7 B_2_) and from ON CBC to ON RGC (Fig. 7 B_5_), although inhibitory inputs from AII AC to OFF RGC were maintained (Fig. 7 B_3_).

Finally, to mimic the effects of gap junction blockers, we set g_gap junction_ to 0 nS. This manipulation reduced membrane oscillations in AII AC (Fig. 7 C_3_) and rendered inhibitory inputs from AII AC to OFF CBC and OFF RGC tonic (Fig. 7, C_2_ and C_3_). This resulted in reduced periodicity of OFF RGCs (Fig. 7 C_1_). Furthermore, membrane oscillations in both ON CBC and AII AC disappeared, and tonic excitatory inputs from ON CBC to ON RGC (Fig. 7 C_5_) led to repetitive firing (Fig. 7 C_4_). These simulations demonstrate that our *Trpm1* KO circuit model successfully reproduced the blocker effects comparable to our electrophysiological results (Fig. 2).

## Discussion

In animal models of RP, including the *rd1* mouse, severe malformations in the inner retina complicate our understanding of the mechanisms underlying pathological RGC oscillations. However, the *Trpm1* KO mouse retina, which retains intact photoreceptors, also displays pathological RGC oscillations. Thus, the *Trpm1* KO mouse retina provides a valuable system for investigating the mechanisms of RGC oscillations in a structurally less compromised context.

In the present study, we investigated the mechanisms underlying RGC oscillations in the *Trpm1* KO mouse retina. We found that the RGC oscillations in OFF αRGCs are driven by inhibitory inputs mediated by glycine receptors, while oscillations in ON αRGCs arise from excitatory inputs mediated by glutamate receptors (Figs. 1 and 2). Notably, a gap junction blocker eliminated oscillations in both RGC types, suggesting a common upstream origin within an electrically coupled network (Fig. 2). Furthermore, *Trpm1* KO mouse retina displayed morphological alterations in RBCs, similar to the *rd1* mouse retina (Fig. 3). The membrane potential of RBCs in the *Trpm1* KO mouse retina was significantly hyperpolarized (Fig. 4). Finally, our mathematical modeling reproduced the RGC oscillations by incorporating these structural changes and the loss of ON signals, suggesting that TRPM1 loss, hyperpolarization, and axon shortening are precursors to oscillations in the AII AC network (Figs. 5 and 6).

### Mechanisms of RGC oscillations in the *Trpm1* KO mouse retina

Computer simulations suggest that RGC oscillations in the *Trpm1* KO mouse retina arise from reducing inputs from RBC and ON CBC to AII AC (Figs. 5 and 6). This reduction may be ascribed to shrinkage of RBC axon terminals, which may be caused by the loss of TRPM1 localization at the dendritic tips of RBCs (Fig. 3 N) (Kozuka et al., 2017; Takeuchi et al., 2018). Such smaller RBC axon terminals may impair the functionality of the release machinery, for instance, through a reduction of Ca^2+^ channels and/or a decreased number of ribbons and synaptic vesicles. Therefore, future detailed experiments focusing on the synaptic transmission from BCs (particularly RBCs) to AII ACs are warranted.

Furthermore, the loss of TRPM1 localization at the dendritic tips of ON BCs may lead to their hyperpolarization. Indeed, our data show that the resting membrane potential of RBCs in the *Trpm1* KO mouse retinas is more hyperpolarized than that in the WT mouse retinas (Fig. 4). This result suggests that ON CBCs may also be hyperpolarized in the *Trpm1* KO mouse retina. These changes could, in turn, contribute to a modest hyperpolarization and membrane oscillations of AII ACs.

### Comparison between *Trpm1* KO and *mGluR6* KO mouse retinas

The *Trpm1* KO mouse retina exhibits prominent spontaneous RGC oscillations. In contrast, *mGluR6*-mutant mice, one of CSNB models, exhibit infrequent or absent RGC oscillations (Rentería et al., 2006; Takeuchi et al., 2018; Hasan et al., 2020; Hölzel et al., 2023). For instance, Hasan et al. (2020) found that only ∼4% of RGCs in *mGluR6* KO mice oscillate in darkness. Hölzel et al. (2023) also reported that oscillatory activity can be evoked by light stimulation in a small proportion of RGCs (∼5%), but absent oscillations are observed in the dark. These infrequent or stimulus-dependent oscillations differ markedly from the robust and spontaneous oscillations observed in *Trpm1* KO mouse retinas, where ∼50% of RGCs exhibit oscillatory activity even in the absence of light stimulation (Takeuchi et al., 2018).

In addition to functional differences, the *mGluR6* KO retina also exhibits distinct morphological features compared with the *Trpm1* KO mouse retina. For example, TRPM1 localization at the ON BC dendritic tips is reduced but still detectable in the *mGluR6* KO mouse retinas (Fig. 3 N) (Xu et al., 2012), and the distribution and size of RBC axon terminals are comparable to those in WT mouse retinas (Fig. 3, A–C) (Kozuka et al., 2017; Takeuchi et al., 2018). These observations suggest that the synaptic transmission from RBC to AII AC may be relatively unimpaired in the *mGluR6* KO mouse retina.

Although the loss of mGluR6 is theoretically expected to render TRPM1 channels constitutively open, RBCs in both *Trpm1* KO (Fig. 4, A and B) and *mGluR6* KO mouse retinas are hyperpolarized (Xu et al., 2012). We measured a 0.20 ratio between the slope conductance in the voltage range around the resting membrane potential in the *Trpm1* KO and WT, consistent with a marked reduction in membrane conductance (*Trpm1* KO: 0.17 ± 0.05 nS; WT: 0.86 ± 0.13 nS) (Fig. 4 D). In comparison, Xu et al. (2012) found a ratio of 0.59 for *mGluR6* KO versus WT mice under similar recording conditions (*mGluR6* KO: 1.09 ± 0.08 nS; WT: 1.84 ± 0.26 nS). This difference suggests that the residual TRPM1 channels at the dendritic tips of RBCs in the *mGluR6* KO mouse retina may remain functional and exhibit spontaneous gating. This preserved channel activity may contribute to the structural maturation of RBCs in the *mGluR6* KO mouse retina, thereby explaining their near-normal morphology and a lack of robust network oscillations.

### Comparison between *Trpm1* KO and *rd* mouse retinas

The neural oscillations in the *Trpm1* KO mouse retina revealed in this study share striking similarities to the phenomenon reported in the *rd1* mouse retina. This suggests that despite their distinct genetic backgrounds, both mouse retinas form a functionally equivalent pathological oscillatory network.

The fundamental physiological characteristics of the oscillations are almost identical in both mouse models. The oscillation frequency determined from the extracellular potential of RGCs was ∼8 Hz in the *Trpm1* KO mouse retina (Takeuchi et al., 2018), which is comparable to the ∼9 Hz in the *rd1* mouse retina (Menzler and Zeck, 2011). Furthermore, the phase relationships of oscillations between αRGCs (anti-phase between different-type cell pairs, in-phase between same-type cell pairs) and the pattern of synaptic inputs, in which OFF αRGCs receive oscillatory inhibitory inputs and ON αRGCs receive excitatory inputs (Fig. 1), were in agreement with reports from the *rd1* mouse retina (Margolis et al., 2008, 2014). This convergence of multiple physiological features strongly suggests that a common underlying circuit is in operation.

These functional similarities appear to stem from shared functional and morphological alterations in RBCs. Although RBCs in the *rd1* mouse retina were predicted to be depolarized due to a decrease in mGluR6 (Strettoi and Pignatelli, 2000; Strettoi et al., 2002, 2003), direct recordings have shown that they are hyperpolarized (Borowska et al., 2011), similar to the *Trpm1* KO mouse retina (Fig. 4). This phenomenon is consistent with the marked reduction of TRPM1 at the dendritic tips of RBCs in the *rd1* mouse retina (Fig. 3 N) and aligns with previous reports establishing a link between TRPM1 reduction and RBC hyperpolarization (Ou et al., 2015). Moreover, our study reveals that RBCs in *Trpm1* KO and *rd1* mouse retinas share morphological alterations, including reduced TRPM1 localization at dendritic tips, changes in axonal terminal distribution, and shrinkage of axonal terminals (Fig. 3) (Takeuchi et al., 2018). These shared pathologies likely serve as a critical trigger for driving the downstream oscillatory network.

Taken together, the multiple commonalities, including the oscillation frequency, phase relationships, synaptic input patterns, and the upstream hyperpolarization and morphological alterations in RBCs, strongly support the view that the oscillations in the *Trpm1* KO and *rd1* retinas are essentially equivalent phenomena driven by a common circuit mechanism.

### Retinal oscillations in CSNB and *rd* mouse retinas

*Trpm1* is one of the genes responsible for CSNB (Nakamura et al., 2010). Other genes implicated in CSNB include *mGluR6* and *Ca*_*v*_*1.4*. Additionally, genes such as *Nyx* and *Lrit3*, which encode proteins essential for the correct localization of TRPM1 to the dendritic tips of ON BCs, are also associated with CSNB (Pearring et al., 2011; Neuillé et al., 2015; Zeitz et al., 2015). With the notable exception of the *mGluR6* KO mouse retinas, which retain TRPM1, spontaneous RGC oscillations comparable to those in the *Trpm1* KO mouse retinas have been described in the *Nyx*^*nob*^, *Ca*_*v*_*1.4* KO, and *Lrit3*^*emrgg1*^ mouse retinas (Winkelman et al., 2019; Hasan et al., 2020; Hölzel et al., 2023). RGC oscillations have also been reported in the *rd10* mouse retinas (Goo et al., 2011; Toychiev et al., 2013), other RP model mice. Immunohistochemical investigation, including our findings for the *Trpm1* KO and *rd1* mouse retinas, has revealed that TRPM1 localization is absent or reduced at the dendritic tips of ON BCs in the oscillated retinas, such as *Trpm1* KO, *Nyx*^*nob*^, *Lrit3*^*emrgg1*^, *Ca*_*v*_*1.4* KO, *rd1*, and *rd10* mouse retinas (Fig. 3 N and Table 2) (Koike et al., 2009; Morgans et al., 2009; Križaj et al., 2010; Pearring et al., 2011; Gayet-Primo and Puthussery, 2015; Hasan et al., 2019; Maddox et al., 2020). The *rd1* and *rd10* mice are models of RP that cause photoreceptor degeneration but relatively preserve BCs. This raises the question of why the *rd* retina loses TRPM1 from the dendritic tips of ON BCs despite no direct genetic defect in TRPM1 itself. As Hasan et al. (2019) have shown that LRIT3 is necessary for the localization of TRPM1 to the dendritic membrane of ON BCs and is located at photoreceptor terminals, it is plausible that the extent of photoreceptor degeneration affects LRIT3 expression and/or localization, which may influence the degree of TRPM1 loss at the dendritic tips of RBCs. Collectively, our results suggest that the loss of TRPM1 localization at RBC dendritic tips may precede and contribute to the observed alterations in RBC axon terminal distribution and shrinkage. Therefore, TRPM1 loss could also contribute to RGC oscillations in the *rd* mouse retinas.

**Table 2.** Summary of pathological oscillations and TRPM1 localization in the CSNB and *rd* mouse retinas

Mutant name	Pathological oscillations	Localization of TRPM1	Disease
*Trpm1* KO	Yes, Fig. 1; Takeuchi et al., 2018	Absent, Koike et al., 2009; Morgans et al., 2009	CSNB
*mGluR6* KO	Nearly absent, Takeuchi et al., 2018 Infrequent, Hasan et al., 2020	Slightly weak, Fig. 3 N; Xu et al., 2012	CSNB
*Nyx* ^*nob3*^	Yes, Winkelman et al., 2019; Hölzel et al., 2023	Absent, Pearring et al., 2011	CSNB
*Lrit3* ^*emrgg1*^	Yes, Hasan et al., 2020	Absent, Hasan et al., 2019	CSNB
*Ca*_*v*_ *1.4* KO	Yes, Hölzel et al., 2023	Reduced and/or absent, Maddox et al., 2020	CSNB
*rd1*	Yes, Margolis et al., 2008; Margolis et al., 2014; Stasheff, 2008; Borowska et al., 2011; Menzler and Zeck, 2011; Yee et al., 2012; Choi et al., 2014; Poria and Dhingra, 2015	Reduced, Fig. 3 N; Križaj et al., 2010	RP
*rd10*	Yes, Goo et al., 2011; Toychiev et al., 2013	Absent, Gayet-Primo and Puthussery, 2015	RP

In the degenerating *rd10* mouse retina, it is reported that the membrane potential of RBCs is depolarized when some rod photoreceptors still survive (Li et al., 2024, *Preprint*), suggesting that some TRPM1 channels may remain functional in RBC dendrites during this stage. However, after complete rod photoreceptor degeneration in the *rd10* mouse retina, TRPM1 localization at RBC dendrites is absent. Furthermore, RGC oscillations have also been observed at this stage of degeneration (Goo et al., 2011; Gayet-Primo and Puthussery, 2015). Our proposed mechanism involving TRPM1 loss at RBC dendritic tips as a driver of pathological oscillations may also explain the oscillations observed in the late stage of degeneration in the *rd10* mouse.

It has been reported that the retinoic acid (RA) level increases in the *rd* mouse retinas due to photoreceptor degeneration, which, in turn, contributes to RGC hyperactivity (Telias et al., 2019, 2022). Given that the outer retinal layers remain intact in the *Trpm1* KO retina, it is unlikely that RA levels are elevated in this model. In our present study, we identified common morphological alterations in RBCs from both *Trpm1* KO and *rd1* mouse retinas, including altered terminal distribution in the IPL, reduced axon terminal size, and loss of TRPM1 at dendritic tips. The absence or reduction of TRPM1 localization at RBC dendritic tips is a consistent feature across oscillating retinas, including those in RP models and certain CSNB models. Taken together, these results suggest that the loss of TRPM1 at the dendritic tips of ON BCs could be a significant contributor to RGC oscillations, potentially independent of RA signaling.

### Comparison between computational models

**Figure 8.**
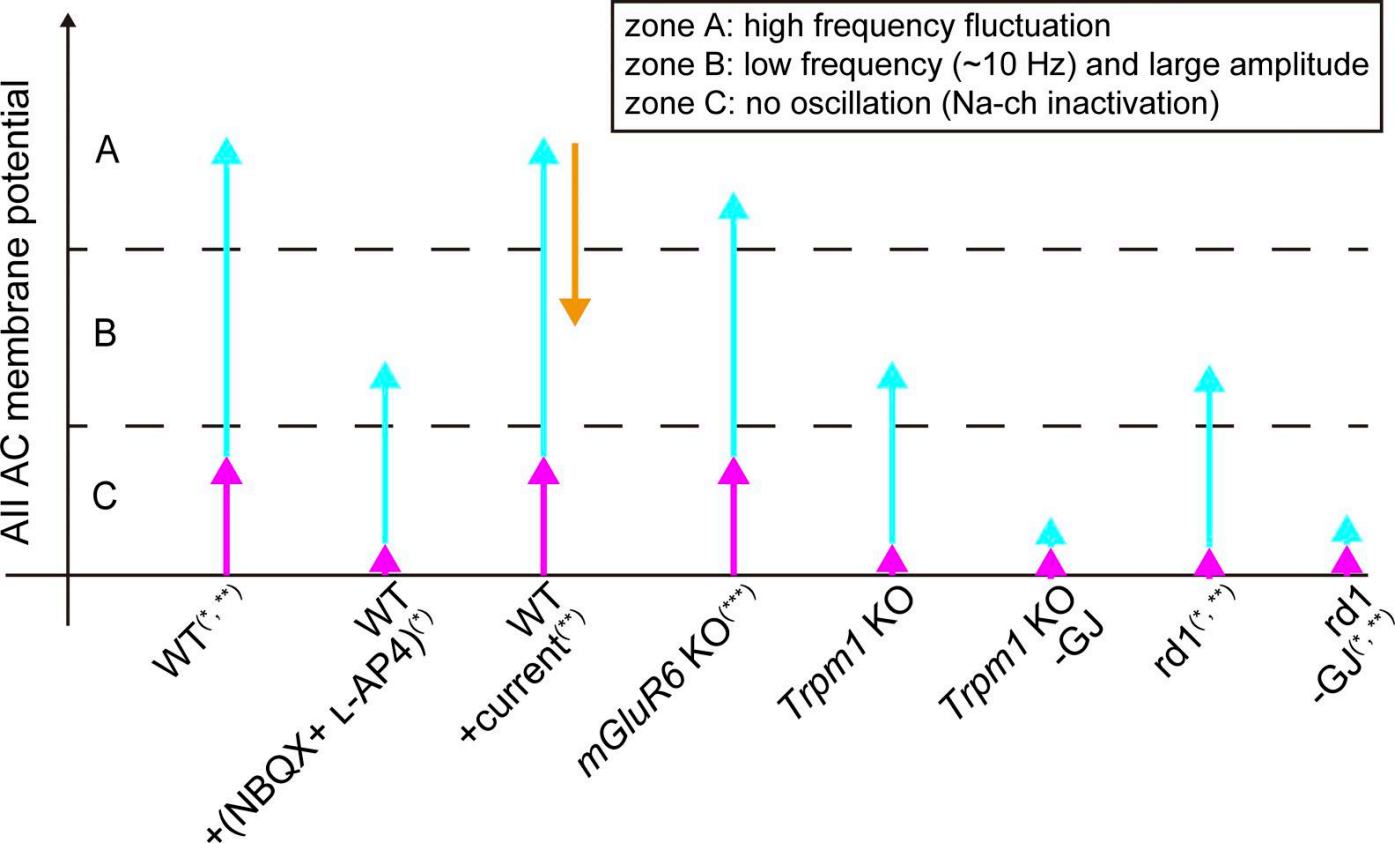
**Relationship between AII AC input and AII AC oscillation.** The AII AC receives excitatory inputs from two primary sources: RBC (magenta) and ON CBC via gap junction (cyan). The orange arrow represents an applied current. GJ, gap junction. The symbols correspond to the following references: *, Trenholm et al., 2012; **, Choi et al., 2014; **, Takeuchi et al., 2018.

Several models have been proposed to account for spontaneous oscillatory activity in the mouse retina, including models for *rd1* retina (Ly et al., 2022) and for CSNB retina (Hölzel et al., 2023). These models, as well as our model, share the common mechanism based on the properties of AII ACs reported by Choi et al. (2014). Fig. 8 presents a conceptual framework that synthesizes our interpretation of experimental results under various conditions (Fig. 2 in this paper; Trenholm et al., 2012; Choi et al., 2014; Takeuchi et al., 2018), illustrating the proposed relationship between the level of AII AC membrane potential and contributing inputs to AII AC. AII AC exhibits three distinct activity modes: high-frequency fluctuations caused by abundant sodium channels at the initiation site in the depolarized state (Fig. 8, zone A), low-frequency (∼10 Hz) and large-amplitude oscillations in the intermediate range of membrane potential (Fig. 8, zone B), and no oscillations due to sodium channel inactivation in the hyperpolarized state (Fig. 8, zone C). Since the low-frequency oscillations in the intermediate range (zone B) are regarded as the source of spontaneous oscillatory activity observed in degenerated retinas, and since the membrane potential of AII AC in the WT mouse retina is more depolarized than that in the *rd1* mouse retina (Choi et al., 2014), AII ACs in the WT mouse retina are presumed to stay in the depolarized state (zone A). Therefore, AII ACs are thought to receive depolarizing inputs from other cell types, and a reduction in these inputs can cause oscillations in AII ACs, leading to spontaneous RGC oscillations. The assumptions of these models are primarily different in regard to the inputs to AII AC. Although other (or previous) models assumed only the gap junctional input from ON CBC to AII AC, our present model considers excitatory inputs from RBCs to AII ACs in addition to the gap junctional input. Considering two excitatory inputs to AII AC enables us to understand physiological results more consistently.

In conclusion, our study suggests a final common mechanism for pathological retinal oscillations, applicable to conditions ranging from severe degeneration to specific genetic alterations like the *Trpm1* KO. This relatively simple model, capable of producing a full range of oscillatory behaviors, provides a robust framework for future investigations into secondary dysfunctions, such as pathological RGC oscillations in various retinal diseases.

## Supplementary Material

Table S1 shows parameter values of synapse models.

Table S2 shows connectivity between cells and synaptic conductance.

## Data Availability

The data that support the findings of this study are available from the corresponding author upon reasonable request.
