# Supplementary material for: A mechanism for pathological oscillations in mouse retinal ganglion cells in a model of night blindness
Source: J Gen Physiol. 2025 Oct 16;157(6):e202413749. doi: 10.1085/jgp.202413749 (PMC12530179; doi:10.1085/jgp.202413749)
Supplement: Table S1 — shows parameter values of synapse models. [file jgp_202413749_tables1.docx]

Table S1. **Parameter values of synapse models**

| ribbon synapse | | conventional synapse | |
| --- | --- | --- | --- |
| parameter | value | parameter | value |
| τ_1A_  τ_A3_  τ_32_  τ_21_  *P*_1, max_  *P*_2, max_  α  β  *V*_th_  *V*_slp_ | 2.0 [ms]  10000 [ms]  2000 [ms]  28 [ms]  0.01  0.04  1.0 [1/ms]  1.1 [1/ms]  −40 [mV]  10 [mV] | *U*  τ_E_  τ_R_  *V*_th_  *V*_slp_ | 0.4  2.7 [ms]  800 [ms]  20 [mV]  −40 [mV] |
