# Supplementary material for: A mechanism for pathological oscillations in mouse retinal ganglion cells in a model of night blindness
Source: J Gen Physiol. 2025 Oct 16;157(6):e202413749. doi: 10.1085/jgp.202413749 (PMC12530179; doi:10.1085/jgp.202413749)
Supplement: Table S2 — shows connectivity between cells and synaptic conductance. [file jgp_202413749_tables2.docx]

Table S2. **Connectivity between cells and synaptic conductance**

|  | **Post** | | | | | | |
| --- | --- | --- | --- | --- | --- | --- | --- |
| **Pre** |  | RBC | ON CBC | OFF CBC | AII AC | ON RGC | OFF RGC |
|  | RBC | - | - | - | Ex (1.2)  126:1 | - | - |
|  | ON CBC | - | gap (0.72) | - | gap (0.5)  16:1 | Ex (13.0)  16:1 | - |
|  | OFF CBC | - | - | gap (0.72) | - | - | Ex (13.0)  16:1 |
|  | AII AC | - | gap (0.5)  14:1 | Inh (0.1)  28:1 | gap (0.08)  all to all | - | Inh (3.0)  14:1 |
|  | ON RGC | - | - | - | - | - | - |
|  | OFF RGC | - | - | - | - | - | gap (1.0)  all to all |

unit in [nS], Ex: excitatory synapse, Inh: inhibitory synapse, gap: gap junction
